# Construction of intelligent moving micro/nanomotors and their applications in biosensing and disease treatment

**DOI:** 10.7150/thno.81845

**Published:** 2023-05-15

**Authors:** Yingshu Guo, Dan Jing, Shiwei Liu, Quan Yuan

**Affiliations:** 1 Shandong Provincial Key Laboratory of Molecular Engineering, School of Chemistry and Chemical Engineering, Qilu University of Technology (Shandong Academy of Sciences), Jinan 250353, P. R. China.; 2 Molecular Science and Biomedicine Laboratory, State Key Laboratory of Chemo/Biosensing and Chemometrics, College of Chemistry and Chemical Engineering, Hunan University, Hunan University, Changsha, 410082, P. R. China.

**Keywords:** micro/nanomotor, biosensing, disease treatment, targeted movement

## Abstract

Micro/nanomotors are containers that pass through liquid media and carry cargo. Because they are tiny, micro/nanomotors exhibit excellent potential for biosensing and disease treatment applications. However, their size also makes overcoming random Brownian forces very challenging for micro/nanomotors moving on targets. Additionally, to achieve desired practical applications, the expensive materials, short lifetimes, poor biocompatibility, complex preparation methods, and side effects of micro/nanomotors must be addressed, and potential adverse effects must be evaluated both* in vivo* and in practical applications. This has led to the continuous development of key materials for driving micro/nanomotors. In this work, we review the working principles of micro/nanomotors. Metallic and nonmetallic nanocomplexes, enzymes, and living cells are explored as key materials for driving micro/nanomotors. We also consider the effects of exogenous stimulations and endogenous substance conditions on micro/nanomotor motions. The discussion focuses on micro/nanomotor applications in biosensing, treating cancer and gynecological diseases, and assisted fertilization. By addressing micro/nanomotor shortcomings, we propose directions for further developing and applying micro/nanomotors.

## 1. Introduction

Power units are the most distinctive features essential to the structure and operation of micro/nanomotors, which are frequently delivered through the bloodstream to target organs or tissues when employed *in vivo* for biosensing or treating diseases. Owing to their extremely small size (and mass), micro/nanomotors undergo severe shear stress in the bloodstream, which increases their rotational velocity and may lower their therapeutic efficacy. To solve this problem, these negative impacts can be mitigated if a motor unit is installed. Thus, targeted drug delivery can be powered by micro/nanomotors to overcome blood-flow resistance 1 and achieve efficient and rapid localization.

In 2002, Whitesides *et al*. developed a chemical-fuel-powered self-propelled plate. Since then, several important advances have been made in the study, generation, and development of micro/nanomotors 2. Nanoparticles (NPs) and their materials have many applications, including the containment and delivery of medicinal products, such as small molecules, biologics, and nucleic acids 3. Recently, micro/nanomotors have enabled various conceivable microoperations and typically comprise fluid-moving, multifunctional nanostructures constructed based on different materials and shapes 4. Recently, research has been continued on the essential elements and circumstances that regulate power in micro/nanomotors. For example, metallic nanomaterials 5, enzymes 6, living cells 7, compound nanomaterials 8, and composite nanomaterials 9,10 can drive micro/nanomotors. Additionally, many natural molecular machines, such as DNA and proteins 11, and many plants exhibit natural drug-carrying structures and simple and effective self-motion mechanisms, which can be combined with other key materials to develop environmental and biomedical applications 12.

An essential component of next-generation drug loading is self-driving carriers that can transport drugs to targets based on specified biomarkers. Such carriers use self-propelled micro/nanomotors and pumps. However, some metals used in self-propelled systems are poisonous to healthy cells and cannot be degraded. Therefore, scientists have developed various micro/nanomotor-driving media. The micro/nanomotor size, favorable biocompatibility 13, orientation and targeting prowess 14, and drug loading have made such motors useful for various applications 15, such as biosensing 16-19 and treating diseases 20-23.

**Figure 1:**
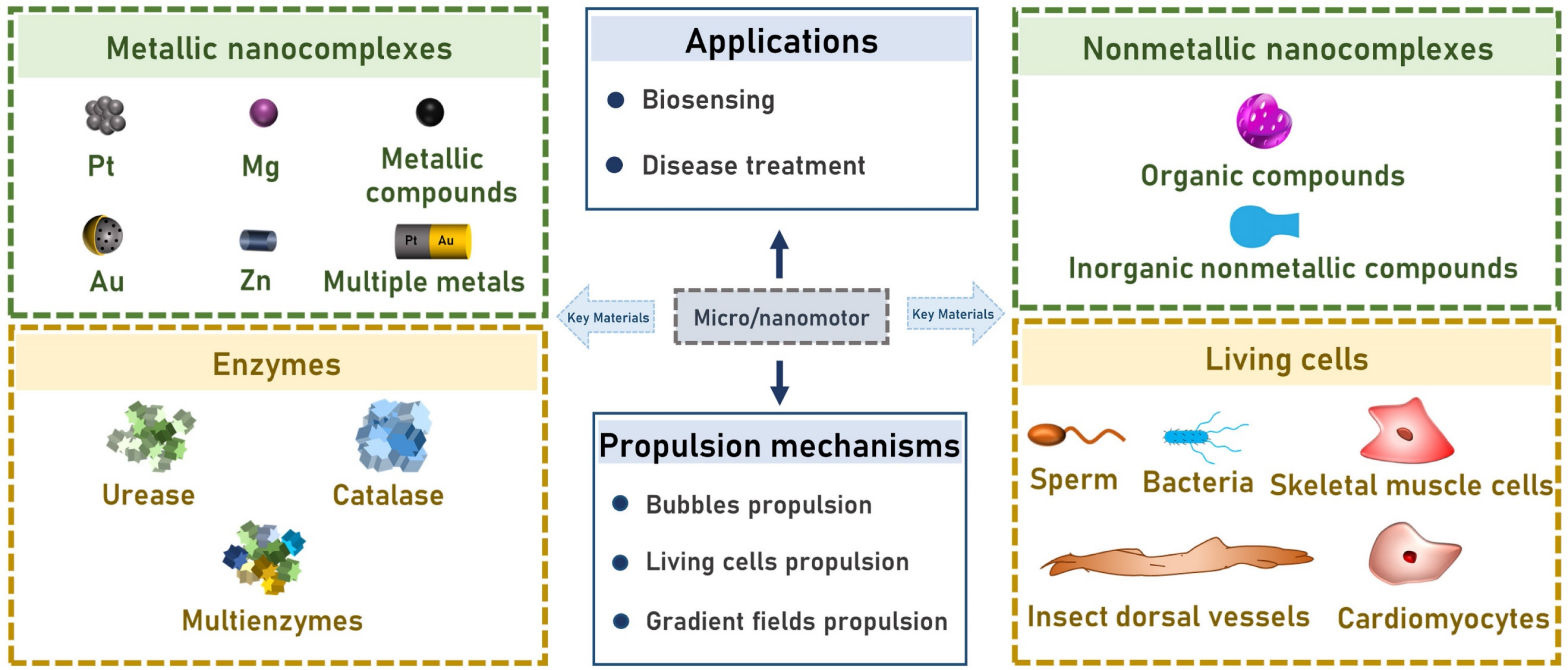
A summary of the classification and and applications for biosensing and disease treatment of micro/nanomotors.

Owing to a wider application range, micro/nanomotor usage has recently become more diversified. Herein, we discuss nanomotors in depth in relation to their key materials and external stimuli, such as light 24-27; magnetism 28-30; sound 31, 32; electricity 33; and endogenous substances, including water 34, urea 35, glucose 36, hydrogen peroxide 37, 38, and gastric acid protons 39-41. The applications of micro/nanomotors in biosensing and treating diseases are discussed in detail, and directions for further research and application prospects of micro/nanomotors are outlined in **Figure 1**.

## 2. Key materials for developing micro/nanomotors

### 2.1. Metallic nanocomplexes

For conventional rockets, the Reynolds number is 10^6^; however, for microrockets, it is just 10^-4^, which is very low. This means that inertia negligibly affects the rocket movement, and instantaneous force usually translates to instantaneous movement. In other words, to move a microrocket continuously in the human body, a continuous driving force must be provided. In addition, for micromotions, the considerable impact of the Brownian motion cannot be ignored. Consequently, the provision of a stable and sustainable propulsion force is the first challenge that researchers have encountered when constructing microrockets 42.

Often, asymmetric product distribution (including gases), which is generated by the redox reaction of the fuel that powers device bodies, functions as the propelling principle for chemically propelled micro/nanorobots. Following the loading of the catalyst onto the micro/nanomotor, redox reactions between the catalyst and fuel produce bubbles. As the bubbles break from the robot body, they generate motion by generating momentum in the opposite direction. An essential component of a bubble-driven tubular micro/nanomotor's overall motion is the bubbles' dynamic evolution. Owing to their rapid velocity and enormous power, such micro/nanomotors have garnered considerable research interest. To catalyze the production of gas from endogenous substances in living tissues or cells, metal NPs have been used as a crucial component of the driving force behind molecular motors. The most typical propulsion mechanisms for micro/nanomotors catalyzed by metals or metal oxides may be bubble propulsion or self-diffusiophoresis. When a metal catalyzes the oxidation of hydrogen peroxide, oxygen is produced by a redox reaction, and the micro/nanomotor advances because of a gradient in the O_2_ concentration. Finite element analysis can be used to model such gradients 43, 44. The inversion symmetry is broken by the Brownian ratchet diffusion mechanism. Without relying on the thermodynamic equilibrium, the directional motion is conceivable in the context of a Brownian ratchet. The developed DNA origami functions as a spinning nanomotor with a ratchet-based driving mechanism 45. The primary materials used for propelling micro/nanomotors and a summary of recent research are listed in **Table 1**.

#### 2.1.1 Pt

A typical micro/nanomotor can be constructed from an inert microsphere semicoated with Pt to accelerate H_2_O_2_ decomposition. The Pt asymmetry decomposes hydrogen peroxide to produce oxygen, which is then used by the Pt catalytic activity to power the micro/nanomotor. Then, Pt is changed asymmetrically through the micro/nanomotor, which is powered by the photothermal conversion.

**Figure 2:**
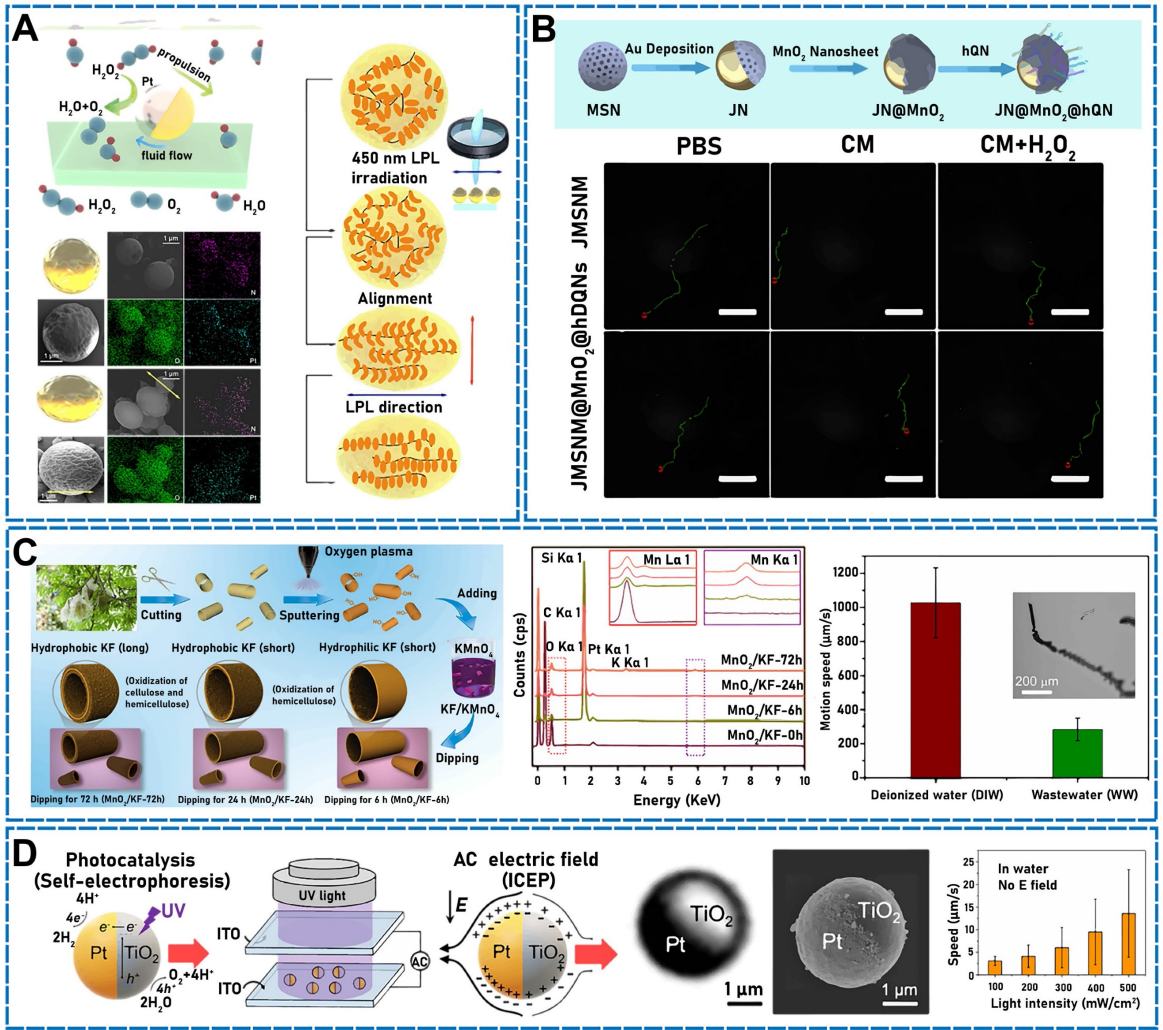
(A) Self-propulsion mechanism of Pt-PAzoMA. Scheme and SEM images of micro/nanomotors with EDS mapping. The deformation mechanism of the micro/nanomotors. Reproduced with permission 47. Copyright 2021, American Chemical Society. (B) Design of JN@MnO_2_@hQN nanoplatform and imaging of miRNAs in Cells. Trajectory states of JN and JN@MnO_2_@hQN. Reproduced with permission 63. Copyright 2021, American Chemical Society. (C) Fabrication of our MnO_2_/KF micro/nanomotors. EDX spectra of the MnO_2_/KF micro/nanomotors. Motion speeds of micro/nanomotors. Reproduced with permission 64. Copyright 2021, American Chemical Society. (D) Working mechanism of the TiO_2_-Pt hybrid micro/nanomotor. Optical micrograph and SEM of a TiO_2_-Pt Janus microsphere. Speeds of a TiO_2_-Pt micro/nanomotor in light. Reproduced with permission 75. Copyright 2020, American Chemical Society.

Hwang *et al*. used asymmetric Pt modification to develop a micro/nanomotor that could remove Cs ions from water by functionalizing silica microspheres with CuFC, depositing magnetic nickel on half of the microsphere surfaces and a Pt catalyst layer on the other half. The Pt on the microsphere surfaces decomposed hydrogen peroxide in the solution, which formed bubbles on the microsphere surfaces and accelerated the micromotion. The metallic Ni layer enabled the micro/nanomotor to be regulated remotely while considerably accelerating the propulsion by stimulating an external magnetic field 46. A similar micro/nanomotor was developed by Díez* et al*. 14 using metallic Pt for asymmetrically changing the surface of mesoporous materials. The mesoporous silica NP (MSNP) face functioned as a drug-loaded nanocontainer, the platinum nanodendrite face functioned as the driving element, and the disulfide-containing oligo (ethylene glycol) chain (S-S-PEG) functioned as the gating mechanism. The nanomotor accelerated the self-propulsion of bubbles by catalyzing the decomposition of low-concentration hydrogen peroxide to turn chemical energy into oxygen. Another team varied the motion of a deformed Pt-PAzoMA Janus micromotor (JM) **(Figure 2A)** comprising homopolymer poly[6-(4-methoxy-4′-oxyazobenzene) hexyl methacrylate] (PAzoMA) particles, wherein half of the particle surfaces were coated with Pt 47. The motion was inspired by the natural landing of flying squirrels. When hydrogen peroxide was employed as fuel, a considerable oxygen gradient was generated on the Pt side of the particles, indicating that self-diffusion electrophoresis functioned based on the propulsion principle. Using the same theory, Chan Woo Park *et al*. asymmetrically deposited metallic Pt to fabricate micro/nanomotors 48. Half of the Pt NPs were deposited on the surface of magnetic illite microspheres so that Pt catalyzed the decomposition of hydrogen peroxide to generate asymmetric bubbles and achieve self-propulsion. The micro/nanomotor exhibited a high velocity of 265 µm/s. In addition, Yeonsoo Lee* et al.* used a self-propelled micro/nanomotor for selectively removing cesium from a high-salt environment by generating asymmetric bubbles through the Pt-catalyzed decomposition of hydrogen peroxide 49.

First, the micro/nanomotor added sulfur to NaX to improve Cs^+^ selectivity by increasing additional Lewis acid-base interactions interactions and then half-deposited on Pt on the S-NaX surface, which catalytically decomposed H_2_O_2_ to push the bubbles. In addition to catalyzing endogenous chemicals, Pt can perform photothermal conversion, which helps nanomotor propulsion. Wan* et al*. produced a mesoporous/macroporous silica (MMS)/platinum (Pt) nanomotors coated with a platelet membrane 50. Owing to the nonuniform distribution of Pt NPs in the system, when the micro/nanomotor was externally stimulated using a near-infrared (NIR) light, the motor generated heat unevenly. Therefore, thermophoresis was the nanomotor driving force 51.

#### 2.1.2 Mg

Hydrogen peroxide, which is hazardous to living tissues and cells, can potentially be replaced by water, which is ubiquitous in organisms and, therefore, may be a perfect energy source. Mg is an active metal that combines with water and H^+^ to produce gas. Several Mg-based micro/nanomotors have been examined and explored by various researchers.

Exploiting the Mg activity, Emil Karshalev* et al*. designed an Mg-based micro/nanomotor oral pill by uniformly dispersing Mg-based micro/nanomotors in a pill matrix 40. A Mg/TiO_2_ poly(lactic-*co*-glycolic acid) (Mg/TiO_2_/PLGA) micro/nanomotor carried the cargo. When the pills reached the strongly acidic gastric environment, they were activated, and gastric juices rapidly decomposed and dissolved the sugar matrix, which released the pill's contents. The engine's magnesium core reacted with the stomach acid to produce hydrogen gas, which produced efficient bubble propulsion. Using the same catalytic decomposition mechanism, Zhang *et al*. combined a Mg-based micro/nanomotor with living macrophage (MΦ) cells to form a unique MΦ-Mg biohybrid motor system 52. When the motor was placed in an acidic environment, the Mg core chemically reacted with protons in the acidic liquid, and the motor generated hydrogen tail bubbles, which provided propulsion. Additionally, Mg is an active metal that can use water as a fuel source and reacts to generate hydrogen and self-propulsion. Wang* et al*. proposed a method for sequentially modifying PLGA, sodium alginate (ALG), chitosan (CHI), and anti-CD3 on the upper surface of Mg microspheres to fabricate anti-CD3-loaded PLGA/ALG/CHI Mg micro/nanomotor systems 53 exhibiting degradable components that were self-propelled by the reaction between Mg and water to generate gas so that the micro/nanomotors exhibited good biological properties and compatibility. Similarly, researchers have produced organic-inorganic hybrid Mg micro/nanomotors for enhanced catalytic activity and synergistic hydrogen chemotherapy 54. Biodegradable micro/nanomotors were prepared utilizing Mg microparticle templates and then asymmetrically coated with PLGA. Magnesium reacted with water to generate enough H_2_ to drive the micro/nanomotor motion and enable enhanced diffusion.

#### 2.1.3 Zn

In addition to Pt and Mg micro/nanomotors, Zn-based ones are frequently used as propellers. The entire motion of bubble-driven tubular micro/nanomotors critically depends on the bubbles' dynamic behavior. Because of their rapid velocity and high power, bubble-driven micro/nanomotors exhibit considerable responsiveness.

With Zn as the key material, micro/nanomotors are propelled by generating air bubbles. Zhou* et al*. used poly(amino acid) to develop a drug-loaded microrocket 55 comprising a Zn-particle core, a thin iron (Fe) middle layer, and a poly(aspartic acid) outer layer. The microrocket was magnetically controlled by adding an iron layer. Owing to its good compatibility and safety, iron has been widely applied as a biomedical material 56. When the microrocket was immersed in a gastric acid simulant, numerous hydrogen bubbles were released and generated self-propulsion. Using the same mechanism, researchers designed a multichambered tubular micro/nanomotor with a cargo compartment protected by a pH-responsive cap at the front end and a zinc motor at the back end to achieve a site-specific response to cargo release and prolong retention in gastric tissue 57. This micro/nanomotor exhibited a powerful push in gastric acid. In an acidic environment, zinc reacted with gastric acid protons, which enabled rapid and autonomous directional actuation. Recently, Cui *et al*. designed a microrod comprising a zinc core and positively charged poly(3,4-ethylenedioxythiophene) (PEDOT^+^) shell containing an anionic model drug dissolved in an electrolyte solution 58. A galvanic cell was formed to construct a battery-operated drug delivery system. When the zinc-based microrod battery was immersed in a physiological environment at a certain pH, both groups of redox reactions proceeded spontaneously, and the reaction progression depended on the pH of the immersion environment. At low pH levels, H^+^ was converted to H_2_, and bubble generation produced a recoil force on the microrod, which caused the micro/nanomotor to operate autonomously. In addition, zinc particles can enhance micro/nanomotor propulsion through the galvanic effect with metal particles. In one study, a Janus gallium/zinc (Ga/Zn) micro/nanomotor exhibiting excellent biodegradability and biocompatibility was developed 59 by asymmetrically coating liquid metallic Ga on Zn particle surfaces. In an acidic solution simulating gastric acid, the hydrogen, which reacted with the zinc and acid, enabled the micro/nanomotor to self-propel up to 383 μm/s by generating air bubbles.

#### 2.1.4 Au

When stimulated by endogenous substances, Au cannot generate bubbles because its catalytic activity is not as powerful as that of Mg and Zn. However, Au does exhibit good heat conversion ability and can be employed as a micro/nanomotor driving mechanism.

By exploiting the good photothermal properties of Au, an NIR photodynamic Janus mesoporous silica nanomotor (JMSNM) micro/nanomotor comprising an MΦ cellular membrane was developed 60. Owing to the asymmetry of the Au half-shell, a photothermal effect was generated when the JMSNM micro/nanomotor was exposed to NIR light, and the thermal gradient generated by the photothermal effect crossed the Janus boundary of the JMSNMs and generated a self-heating kinetic force that pushed the JMSNMs. Through the same mechanism, the asymmetrical distribution of Au could generate photothermal conversion by irradiation with an external light source. Shao* et al*. designed erythrocyte-membrane-encapsulated Janus polymer motors, which self-propelled when exogenously stimulated by NIR light 61. These micro/nanomotors comprised heparin (Hep) and CHI, and the surface was modified by an erythrocyte membrane. The capsule was formed by opposite charges on the surface, and both natural polymers were assembled layer by layer (LbL). To propel the micro/nanomotor under fuel-free conditions, LbL capsules were partially wrapped in gold shells. When the capsules were irradiated using NIR light, a partial thermal gradient was generated owing to the asymmetrical Au modification, which produced a thermophoretic effect that drove the micro/nanomotor motion. Recently, researchers have fabricated NIR-driven JMSNMs 62 by doping MSNPs with gadolinium and depositing Au on the hemispherical NP surface. Again, owing to the asymmetry of the Au half-shell, an asymmetric thermal gradient was generated by irradiation with NIR, and the nanomotor achieved efficient self-propulsion. Using the same photothermal conversion propulsion mechanism, Lin* et al*. designed an NIR light-propulsion Janus-based nanoplatform (**Figure 2B**) 63. Au half-shell-covered Janus nanomotors were loaded with an endophagy adjuvant (MnO_2_ nanosheets) to produce microRNAs (miRNA)-responsive hairpin DNA quadrilateral-nanostructured probes through catalytic hairpin assembly. Surface plasmon resonance absorption generated a thermal gradient on the Au half-shell of the nanoplatform, which promoted thermophoresis.

#### 2.1.5 Metallic compounds

In addition to frequently employed Pt metal particles, some common metallic compounds, such as MnO_2_, can catalytically decompose hydrogen peroxide to generate gas and, therefore, propulsion. Additionally, Fe_3_O_4_'s characteristics enable magnetically controlled propulsion. Owing to its strong photocatalytic characteristics, bismuth oxyiodide is also a key material for application to micro/nanomotors.

In one study, a micro/nanomotor exhibiting double-layered microtubes was designed using electrochemically reduced graphene oxide/MnO_2_ 10. Owing to the rough inner surface and porous structure of the microtubes, the micro/nanomotor exhibited good catalytic activity, which could decompose H_2_O_2_ to generate gas and achieve rapid and efficient propulsion. Chen* et al*. used naturally abundant kapok fibers (KFs) as a template to design a manganese dioxide (MnO_2_)-catalyzed tubular micro/nanomotor (**Figure 2C**) 64 wherein MnO_2_ was deposited and modified on the inner and outer walls of the KFs. Hydrogen peroxide (H_2_O_2_) was catalytically decomposed using MnO_2_, and oxygen (O_2_) bubbles were generated to self-propel the micro/nanomotor. Similarly, Yang *et al*. developed a magnetic micro/nanomotor using natural KFs as a template to uniformly grow laccase-immobilized Fe-BTC (Iron 1,3,5-benzenetricarboxylate) metal organic framework (MOF) NPs on Mn_2_O_3_-NiFe_2_O_4_ nanosheets 65. The micro/nanomotor used the catalytic effect of Mn_2_O_3_ on H_2_O_2_ and moved at 120 μm/s under the action of decomposing gas. By exploiting the excellent photocatalytic properties of bismuth oxyiodide (BiOI), a BiOI-based JM was fabricated 16. The micro/nanomotor could be activated by irradiation with visible light, including blue and green wavelengths in broad-spectrum light, and was developed using only biotechnology. Therefore, the micro/nanomotor could undergo a photocatalytic reaction in pure water without adding any chemical fuels. The micro/nanomotor was propelled, and the photocatalytic driving force was regulated by changing the visible-light power. Additionally, magnetic fields have been used to propel micro/nanomotors. Xie* et al*. developed a strategy for programming hematite colloidal particles into microrobot swarms in liquids, chains, vortices, and ribbons in alternating magnetic fields 66. Peanut-like hematite colloidal particles exhibiting long and short axes of 3 and 2 µm, respectively, could form numerous microrobots. The short axis of the microrobots exhibited permanent momentum. In the absence of external stimuli, the microrobots naturally aggregated into a mass owing to their intrinsic magnetic dipole forces. An external rotating magnetic field could produce circular polarization on any plane in three-dimensional (3D) space, thereby exciting the microrobots to achieve different motions (such as oscillation, swinging, rolling, spinning, and tumbling). In one study, calcium-crosslinked degradable alginate (Ca-Alg) hydrogel capsules were prepared utilizing a hydrodynamically electrospraying ionization jet assisted by low-frequency ultrasound 67. The ultrasound increased the capsule uniformity, and the formation of a gel layer was simultaneously prevented at the air-water interface. The capsules were used to encapsulate model drugs, and monolayer polylactic acid was applied as a sealant. The capsule propulsion was guided by adding superparamagnetic magnetite particles (Fe_3_O_4_) to the capsule and applying a stable or an alternating oscillating magnetic field. By applying an exogenously stimulating magnetic field, Wang* et al*. recently used an easily fabricated and versatile setup to design magnetic helical hydrogel micro/nanomotors (PVA@Fe_3_O_4_@CXCL12) 68. Magnetic ferric oxide NPs were embedded in the hydrogel micro/nanomotor, which enabled the remote operation and actuation of the micro/nanomotor through the magnetic field. The wireless control conveyed biocompatibility to the hydrogel motor. In addition, Li *et al*. designed semiartificial magnetotactic bacteria (SAMTB), which exhibited a tunable magnetic moment and good sensitivity 69. Synthesized Fe_3_O_4_ magnetic NPs (MNPs) were attached to the surfaces of *Magnetospirillum magneticum* AMB-1 (AMB) and magnetotactic bacteria (MTB) at a high usage rate. Fe_3_O_4_ MNPs were mainly connected to AMB-1 through electrostatic interactions. By varying the concentration, size, and surface charge of MNPs, the bulk magnetism of the SAMTB could be manipulated to control magnetic sensitivity and mobility. Wu* et al*. designed a chemical/magnetic hybrid micro/nanomotor based on Fe_3_O_4_ MNPs and GOx-modified cubic calcium carbonate microparticles (CCMPs) 70. The GOx enzymatic reaction functioned as a motor device using both starvation therapy and glucose as fuel, which enabled the hybrid micro/nanomotor to self-propel. Additionally, owing to the incorporation of Fe_3_O_4_ NPs, which played the role of a magnetic motor, the kinetic energy of the micro/nanomotor increased. The device exhibited both chemical and magnetic engines, which drove the motion. Owing to graphene oxide (GO) photocatalytic properties, a cuprous oxide@GO (Cu_2_O@GO) micro/nanomotor was introduced for high-efficiency actuation under different light conditions 71 using various biocompatible fuels (e.g., glucose, leucine, and urine). The addition of GO to the micro/nanomotor enhanced the photocatalytic performance, which enabled efficient propulsion under visible, and even NIR, light.

#### 2.1.6 Multiple metals

In addition to hydrogen peroxide and water, glucose is a relatively common endogenous stimulant in the living body. After decomposition, oxygen is generated to propel micro/nanomotors. Owing to their unique catalytic properties, Au and Pt bind at specific positions in the same nanostructure to achieve synergistic catalysis by colocalizing different active sites. Kwon *et al*. introduced an Au/Pt-based Janus nanostructure resembling an “egg-in-nest” morphology (Au/Pt-Ens) 72. The nested Pt structure was asymmetrical: The bottom was closed and nested by Au NPs, and Au NPs fixed the nested Pt structure in place. The nested morphology and large openings in this nanostructure increased the entry of the fuel (glucose) and mass transfer of the product, and the nanostructure [Au-Pt] active site was at one end of the NP. The concentration gradients at the cavity and outer surface competed to govern the nanostructure mobility. Researchers have previously prepared uricase-powered Janus upconversion NP-SiO_2_ micromotors (JUS motors) by asymmetrically immobilizing enzymes and upconversion NPs on silicon microparticle surfaces 73. Owing to the asymmetrical distribution and bilateral structure of the enzymes, the JUS motors could autonomously move, driven by the uric acid in urine. Because some micro/nanomotor constituent materials are magnetic, propulsion was achieved by applying magnetic fields 74. Iron (Fe) and platinum (Pt) were codeposited to prepare ferromagnetic FePt nanopropellers. The main nanopropeller components were FePt and silica, and the nanopropellers were driven by gradient-free rotating millitesla fields. Owing to their high magnetic moment, the FePt nanopropellers exhibited a propulsion velocity superior to those of both the Fe- and Ni-based magnetic propellers. Light is among the most used exogenous stimuli. Some researchers have found that under the combined action of light irradiation and an electric field, the micro/nanomotor velocity can be greatly improved. Researchers previously designed a TiO_2_-Pt Janus microspheres (**Figure 2D**) to drive a photocatalytic titania-platinum (TiO_2_-Pt) micro/nanomotor under the combined action of light and an alternating current electric field 75. Invariably, the systemic velocity was more rapid than the sum of the individual velocities under any other propulsion.

Although the development of micro/nanomotors has excellent application prospects, several obstacles must still be overcome. The propulsion or application of micro/nanomotors is difficult because the key materials required for fabricating micro/nanomotors are restricted or constrained by the endogenous or external stimulation these materials require. For instance, owing to its excellent photothermal conversion ability, Pt, which is frequently employed in such systems, also has drawbacks. Pt catalysts are expensive, hazardous, unstable, and scarce, which severely limit the application potential of Pt catalysts 76. Similarly, Au NPs are costly. Although Mg exhibits numerous positive inherent properties, numerous important obstacles must be overcome before it can be employed in challenging practical applications. Notably, the rapid consumption of magnesium during actuation and small micro/nanomotors usually result in a short lifespan for Mg-based motors. Numerous measures can be used to extend the lifespan of Mg engines, including reducing the bubble formation frequency by rationally allocating micro/nanomotor compositional characteristics (e.g., upgrading the opening shape and size) or alloying with other metals and reducing the corrosion rate by reducing the number of pitting-induced anions. Additionally, the consideration of how Mg micro/nanomotors release hydrogen bubbles is important. The assessment of this latent negative effect is crucial for *in vivo* applications because the danger from the formation of gas embolisms in blood arteries is considerable owing to the relatively poor solubility of H_2_ in water. Undeniably, excess Mg ions can cause hypermagnesemia, which exhibits symptoms such as nausea, muscle weakness, a low heart rate, and hypotension. Additionally, excess Mg ions can increase the local osmotic pressure, which can disturb cellular homeostasis. Before magnesium micro/nanomotors can be therapeutically applied, these problems must be solved 77. Zn NPs have been used as essential components for manufacturing nanomotors. Zn exhibits high metallic catalytic activity, which enables it to react with gastric juice protons to produce gas and release numerous hydrogen bubbles. However, because Zn-based systems readily corrode, their lifespans are shortened 56. The primary components that power nanomotors fundamentally comprise elementary metallic NPs, which makes such nanomotors both expensive to produce and potentially harmful to living organisms and biological cells. However, these drawbacks can be avoided using porous and mesoporous materials loaded with compound NPs, such as Fe_3_O_4_ or MnO_2_. Fe_3_O_4_ exhibits magnetic properties that enable good control accuracy when utilizing magnetic fields, which can be used to construct nanomotors for accurately delivering drugs. Subsequently, nanomotors can be recycled by applying magnetic Fe_3_O_4_ particles, which are also very biocompatible. MnO_2_ particles decompose hydrogen peroxide (H_2_O_2_) fuel to produce oxygen bubbles and generate the necessary mechanical force for operating motors. MnO_2_ motors are favored for their low cost, simple production, wide range of fuel concentrations, adjustable geometry and size, long lifetime, high efficiency, and programmable crystal structure and surface topography. For water filtration, MnO_2_-based micro/nanomotors play dual roles of catalytic decomposition and adsorption-bubble separation. However, few studies have examined MnO_2_ as a catalyst. Many hybrid micro/nanomotors employ two key materials. Recent developments in hybrid micro/nanomotors have attracted considerable research attention. Considering various energy sources that micro/nanomotors can utilize, hybrid micro/nanomotors activated by multiple power sources can switch between two or more driving forces, which enables motors to accelerate/decelerate, move forward/backward, and/or switch motion modes 75. Composite NPs are prepared by depositing two or more metals, a combination of metal and compound, or two or more compound NPs on special nano/microspheres through a certain method. Alternatively, this means two or more substances are used as key materials for driving micro/nanomotors. Although the cell absorption capacity and movement velocity of composite NPs are higher than those of single-metal NPs 78, such systems exhibit the same problems as those of simple metal NPs. That is, owing to the scarcity of precious metal resources, certain metals are more expensive and can be toxic to living organisms or biological cells. Magnesium-based micro/nanomotors driven by a dual mechanism are propelled by the reaction between Mg and H_2_O. Pt decomposes when it encounters H_2_O_2_, which prolongs the motion life. Owing to the asymmetric Pt coating, the corrosion of the active metal (Mg) is decelerated, and the micro/nanomotor life is prolonged. In addition, the micro/nanomotor exhibits high toxicity and precious metal scarcity 79. Furthermore, compared with Au and Ag NPs, Fe ones are feasible for driving micro/nanomotors. Moreover, micro/nanomotor driving requires the use of H_2_O_2_ as a fuel source, which is toxic to living cells. However, instead of using H_2_O_2_, Au/Pt-Ens can oxidize glucose and generate O_2_ gas, which reduces the toxicity to living cells and organisms 72. Additionally, Au/Pt-Ens can overcome stochastic Brownian forces while imparting lifelike directed mechanical motion; is biologically nontoxic; and uses plentiful fuels, such as glucose. However, the fabrication of such micro/nanomotors is costly and requires the research and development of inexpensive metals to achieve the same effect. Ferromagnetic FePt nanomotors, which are noncytotoxic and biocompatible, exhibit a remanence and magnetization comparable with those of permanent NdFeB micromagnets 74. Magnetic particle systems are particularly promising because they can be manipulated by magnetic fields. The face-centered tetragonal (fct) L1_0_ phase of FePt is a promising magnetic material, which is noncytotoxic, nonimmunogenic, and highly biocompatible and exhibits a strong magnetic residue. FePt is a chemically stable rare-earth-free permanent magnetic material exhibiting high magnetic crystal anisotropy. FePt NPs exhibit high remanent magnetization, saturation, energy production, and coercivity. Chemical/magnetic hybrid micro/nanomotors can be fabricated using Fe_3_O_4_ MNPs and GOx-decorated CCMPs 70. Fe_3_O_4_ NPs were incorporated into a hybrid micro/nanomotor intended to function as a magnetic motor and not only enhanced the kinetic energy of the device but also controlled the motion direction. The motor was propelled by glucose, a typical bioavailable and biocompatible fuel, and was activated by a magnetic field. The magnetic and chemical engines both improved motor behavior, provided propulsion, and enhanced cellular uptake. Simultaneously, the GOx-initiated biocatalytic reaction consumed intracellular glucose, which further reduced cancer cell viability. Additionally, Fe_3_O_4_ is economically feasible. Cu_2_O@GO micro/nanomotors featured localized light-induced thermal effects and enhanced photocatalytic performance; that is, this motor could be operated under NIR irradiation with a broad range of biocompatible fuels, such as leucine, glucose, and urea solutions 71. For this photocatalytic micro/nanomotor, the excitation wavelength extended to NIR light. This strategy for improving the performance of light-driven micro/nanomotors by GO doping opens an avenue for fabricating micro/nanomotors showing excellent potential for bioenvironmental applications.

### 2.2 Nonmetallic nanocomplexes

#### 2.2.1 Inorganic nonmetallic compounds

In contrast to metallic nanocomposites, inorganic nonmetallic compounds frequently appear in micro/nanomotors that rely on the photothermal conversion of essential components under exogenous light stimulation. For instance, GO and g-C_3_N_4_ are photocatalytic materials that enable photothermal conversion to power micro/nanomotors in electromagnetic fields.

**Figure 3:**
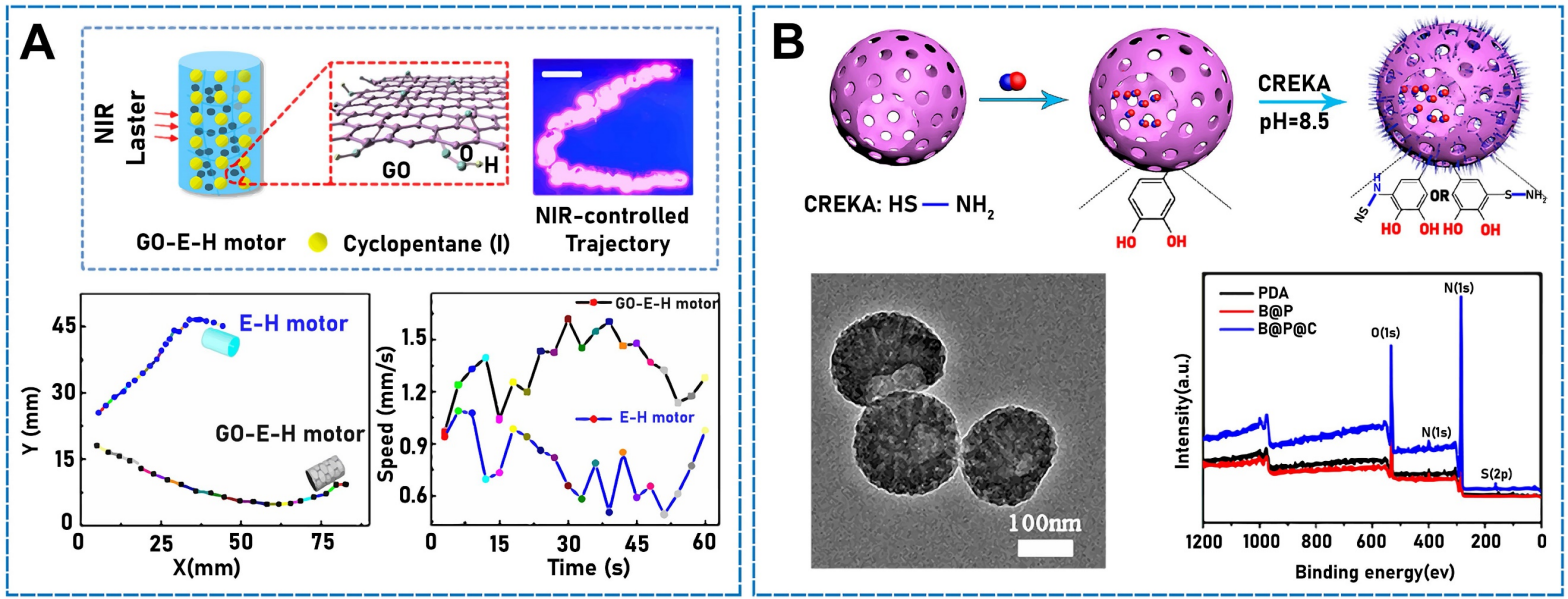
(A) The composition of GO-E-H-motor. The trajectories of a GO-E-H-motor. The velocity of a GO-E-H-motor. Reproduced with permission 80. Copyright 2017, American Chemical Society. (B) Preparation process of B@P@C nanosweeper. TEM images of B@P@C nanosweepers. XPS of PDA, B@P, and B@P@C. Reproduced with permission 88. Copyright 2021, American Chemical Society.

Oil-in-water emulsion templating, adopted by Wang* et al*., was sufficiently simple and versatile to fabricate an emulsion-hydrogel motor comprising a hydrogel matrix and low-boiling-point fuel-oil emulsion 80. Under stimulation, the thermally induced phase transition can generate bubbles that are violently ejected from one side of the motor to achieve efficient propulsion. Such motors can be integrated with Fe_3_O_4_ NPs for magnetic guidance. Under NIR light conditions, the addition of GO (**Figure 3A**) can control the distance and accurately regulate the motion of motors, including the orientation, velocity, and trajectory. Additionally, reduced-GO aerogel microspheres can function as power devices 81. In that study, microspheres were produced using electrospray ionization, which is simple to operate, and the systems exhibited size controllability and an isotropic structure. In water, graphene can convert light to heat in an externally stimulated asymmetric light field and drive a micro/nanomotor because of the asymmetric thermal gradient generated in water. Furthermore, g-C_3_N_4_ is a promising photocatalytic material characterized by a metal-free structure, good thermochemical stability, and visible-light responsiveness 82. Dong* et al*. combined g-C_3_N_4_ photocatalysts and self-propelled jet carbon micro/nanomotor carbon bottles utilizing infrared light and active mass transfer to enhance the photocatalytic performance of the micro/nanomotor 83. When stimulated by light, the combination exhibited improved motility and excellent self-propulsion.

#### 2.2.2 Organic compounds

Numerous chemical molecules require exogenous stimulation for propelling motors. Although some chemicals are micro/nanomotor components, they may also be employed as raw materials for driving those micro/nanomotors, and micro/nanomotors can be promoted by producing gas.

Inspired by the well-established model of dye-sensitized solar cells, light-driven artificial microfilaments can be produced and derive energy from visible light, which enables propulsion and navigation under visible-light conditions. Zheng* et al*. designed and developed a motor that absorbed visible-light photons when a photosensitizer (organic dye) was loaded into titanium dioxide, and electrons were injected from it into titanium dioxide nanowires, which generated a propulsive photocurrent 84. In addition to their photothermal conversion properties, some organic compounds can be used as raw materials to drive micro/nanomotors while simultaneously being used as micro/nanomotor components. An NO-gas-driven nanomotor originally comprising hyperbranched polyamide/L-arginine has previously been developed 85. The micro/nanomotors comprised endogenous biochemical reactions in living organisms, which converted the amino acid L-arginine to nitric oxide (NO) in the presence of NO synthase (NOS) or reactive oxygen species (ROS). Some organic compounds can be stimulated using sound fields. Ye *et al*. found that a Janus mesoporous SiO_2_ nanorod partially coated with gold (Au NR-mSiO_2_) deeply penetrated tumor tissue under exogenous stimulation with ultrasound 86. When 2,2-azobis[2-(2-imidazolin-2-yl) propane] dihydrochloride (AIPH) was ultrasonicated in mSiO_2_ (Au NR-mSiO_2_/AIPH), a high-efficiency N_2_ microbubble developed and drove the nanomotor. Air bubbles (such as O_2_, H_2_, and NO) can be used as the propulsion power source and play an indispensable role in physiological activities 87. Deng* et al*. designed a hollow asymmetric polydopamine (PDA) NP and modified it with the Cys-Arg-Glu-Lys-Ala (CREKA) peptide, namely, BNN6@PDA@CREKA (B@P@C), to form a donor for loading NO (**Figure 3B**) 88. The resulting micro/nanomotors efficiently aggregated at thrombus sites 89. The micro/nanomotor absorbed NIR light, and the release of NO bubbles rapidly moved the micro/nanomotor and enabled the deep penetration of thrombi by transferring energy to the NO donor (BNN6 (*N*,*N*′-di-sec-butyl-*N*,*N*′-dinitroso-1,4-phenylenediamine)) to achieve a thrombolytic effect. C_3_N_4_ is a common inorganic polymer photocatalyst, which can photocatalytically hydrolyze water. In one study, a visible light-driven micro/nanomotor comprising binary photocatalytic carbon nitride (C_3_N_4_) and photothermal polypyrrole NPs (PPyNPs) was developed under fuel-free conditions 90. Owing to the excellent photocatalytic properties of C_3_N_4_ and photothermal effect of PPyNPs, this micro/nanomotor was driven by two independent but competing mechanisms, namely, self-diffusion and autothermal electrophoreses.

To advance micro/nanomotors, photothermal conversion must typically be induced under the influence of external light. Without the use of any additional chemicals, light is transformed into mechanical energy throughout the process 16. Although infrared light can comprise over 50% of solar radiation, it cannot directly initiate photocatalytic activity owing to its low photon energy 91. Because heat can be used to power micro/nanomotors, infrared light exhibits a high photothermal conversion efficiency (over 85%) 92. Reduced GO (RGO) is a typical graphene-based material that exhibits extraordinary qualities, such as high thermal conductivity, a large surface area, and electrical conductivity. These attributes make RGO a comparatively excellent candidate for high-tech micro/nanomotor applications. Additionally, RGO can efficiently collect NIR photons and quickly convert them to heat energy through the photothermal effect. Previous studies have shown that owing to their porous, multilayered, and hydrophobic structures, RGO-based aerogel microspheres exhibit an exceptional adsorption capacity and ultralow density and might, therefore, be used for treating environmental pollution or delivering medicines. When externally stimulated by asymmetric NIR light, graphene may use photothermal conversion to generate an NIR-induced self-asymmetric thermal gradient in water, which can then be used to power a micro/nanomotor. Owing to its high thermochemical stability, metal-free structure, and visible-light responsiveness, g-C_3_N_4_ is a typical photocatalytic material that shows tremendous application potential. However, g-C_3_N_4_ exhibits a modest hydrogen production efficiency, and its photocatalytic performance must be enhanced using numerous methods. The human body naturally contains organic substances, such as L-arginine and PDA. The metabolic process through which NOS and other ROS convert L-arginine to NO in the human body has garnered interest. L-arginine exhibits immunomodulatory properties that stop the thymus from deteriorating and encourage the development of thymic lymphocytes. However, because hydrogen peroxide is hazardous, an alternative fuel must be found in live cells or organisms. L-arginine can be used as a fuel for tiny, NO-powered micro/nanomotors. The produced NO is used for propulsion and exhibits beneficial effects, such as encouraging endothelialization and anticancer actions. As a fuel, L-arginine does not generate any waste and shows potential for biological applications, such as the treatment of a range of conditions in various tissues, including cancers and blood vessels 85. Similar to L-arginine, PDA transfers energy by absorbing NIR light, which releases NO bubbles. However, owing to its high light-conversion efficiency, the micro/nanomotor functions as a photothermal agent to accelerate the dissolution of blood clots, which results in a combination of mechanical and photothermal thrombolysis 89.

### 2.3 Enzymes

The application of nanozymes (which have capabilities resembling those of enzymes, such as catalase, oxidase, peroxidase, and superoxide dismutase) greatly broadens the scope of microelectromechanical chemical fuels 93. Because they can control velocity with remarkable accuracy, enzyme-based nanomotors are extremely important 94. Nanozyme-driven micro/nanomotors offer a wide range of intriguing applications 95. Enzymes enable conversion between chemical and mechanical energies and are found in numerous organisms. The application of enzymes as biocatalysts to power micro/nanomotors is an important concept. Compared with conventional reaction fuels, fuels used by enzyme-driven micro/nanomotors exhibit superior bioaffinity, good biocompatibility, and considerable potential for biomedical applications. When enzymes are loaded on artificial tubular micro/nanoparticles or spherical particle structures, synthetic enzyme-powered micro/nanomotors (EMNMs) can be catalyzed by enzymes to achieve diverse functions in complex environments 96.

#### 2.3.1 Urease

In enhanced enzyme diffusion and chemotaxis, many enzymes spread more quickly in the presence than in the absence of substrate fuels. Therefore, enzymes can be employed as active molecules 97. Because blood contains urea, urease is a crucial component of molecular motors.

**Figure 4:**
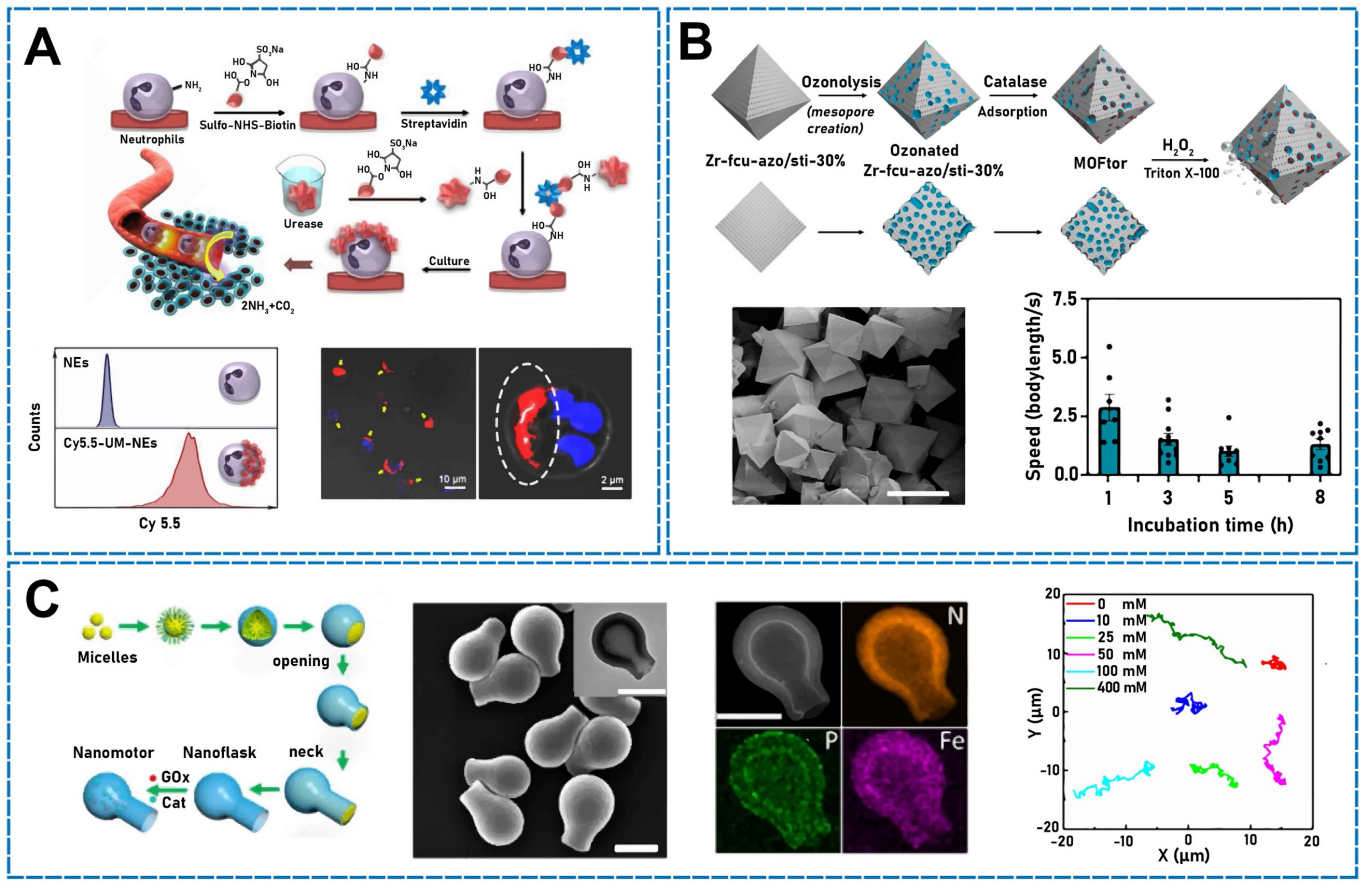
(A) The preparation principle of urease motor. Fluorescence imaging of UM-Cy5.5-Nes. Flow cytometry histograms of UM-Cy5.5-Nes and controlled Nes. Reproduced with permission 1. Copyright 2022, American Chemical Society. (B) Schematic diagram of the synthesis process of MOFtors. FESEM image. Speed of MOFtors at different incubation times. Reproduced with permission 107. Copyright 2020, American Chemical Society. (C) Synthesis of enzyme-loaded round-bottom CNF motors. SEM image of L-CNFs. STEM and EDX mapping images of the enzyme-loaded hydrophilic CNF motors. Trajectories of the hydrophilic CNF motors at different glucose concentrations. Reproduced with permission 109. Copyright 2019, American Chemical Society.

Using silica spheres, Hortelão* et al*. prepared core-shell MSNPs, 3-aminopropyltriethoxysilane-functionalized MSNPs, and MSNP-NH_2_ NPs. The surface exhibited free amino groups, and glutaraldehyde was used as a linking molecule for covalently attaching urease to the NPs and producing urease micro/nanomotors. The urease on the micro/nanomotor surface decomposed urea to ammonia and carbon dioxide, which self-propelled the motors 98. In addition, in a previous study, fiber rods were fabricated using a unique Janus structure and double-sided electrospinning wherein urease and folic acid were conjugated to both sides of Janus rods (JRs) as power sources 99. The urease implantation density determined the movement velocity and trajectory of the JMs, and large mean-squared displacements and high movement velocities were detected in both the extracellular matrix, mimicking tumor tissue, and phosphate-buffered saline. With increasing urease density, the propulsion force also increased. Using endogenous urea stimulation, Tang *et al*. designed a Janus platelet micro/nanomotor, which asymmetrically immobilized urease on the surface of native platelet cells 100. Owing to the asymmetrical distribution of the urease on platelet cells, the urea did not disintegrate uniformly in the biological fluid, which enhanced the chemical electrophoretic motion. Similarly, urease-micro/nanomotor-driven Janus-Nes systems have recently been synthesized by asymmetrically immobilizing urease on the surface of natural Nes followed by loading urokinase (UK) (**Figure 4A**) coupled with Ag NPs (Ag-UK) 1. The urease-catalyzed endogenous urea produced ammonia and carbon dioxide, which propelled the system. In addition to the attachment of urease to NPs, Jiao* et al*. recently developed asymmetrically modified urease in yeast cells 101. With urea as an endogenous stimulus, self-propulsion was achieved owing to the asymmetrical modification of yeast cells.

#### 2.3.2 Catalase

Because hydrogen peroxide is toxic to cells and tissues, the application of catalase to drive nanomotors and achieve self-propulsion has become a very important research topic. For example, in one study, a high-efficiency enzyme-powered micro/nanomotor was designed and fabricated by combining catalase with a PEDOT^+^ and sodium 4-styrenesulfonate)/Au microtube (PEDOT-PSS/Au) 102. The inner surface of the tube (PEDOT-PSS/Au) was assembled in multiple layers. The micro/nanomotor exhibited efficient movement through stimulation with hydrogen peroxide. Using the same propulsion principle, Wang* et al*. fabricated drug-loaded PLGA micro/nanomotors asymmetrically covered with enzymes (in a patch-like distribution) applied by electrospraying and prepared using 1-ethyl-3-(3-dimethylaminopropyl)carbodiimide coupling and then postfunctionalized with catalase 103. A H_2_O_2_ gradient can sustain the directional movement of nanocarriers, including enzymes, toward high-concentration fuels. With increasing fuel amount, the micro/nanomotor velocity also increases. In addition, micro/nanomotors can be asymmetrically grafted by catalase. In a previous study, JMs were prepared by grafting catalase on the side of JRs to power JMs by catalyzing H_2_O_2_ decomposition 104. Catalase (CAT) was covalently bound and immobilized on one side of JRs, and a TLS11a aptamer was covalently bound to JMs. The catalytic reaction of CAT with hydrogen peroxide can propel JMs. In another study, researchers grafted CAT on one side of Janus fiber rods (JFRs) and combined mannose on the other side 105. The CAT decomposed the hydrogen peroxide to generate oxygen bubbles and propel JMs. In contrast to grafting, CAT can also encapsulate JFRs to generate oxygen by decomposing hydrogen peroxide and propel a micro/nanomotor. An ultrasmall-cell (⌀∼150 nm) nanomotor has previously been developed and encapsulated in CAT for application to biocatalysts 106. The device decomposed H_2_O_2_, and the generated O_2_ was used as a power supply. Likewise, Yang* et al*. designed an enzyme-powered porous micro/nanomotor constructed from MOFs, as shown in **Figure 4B** 107. First, presynthesized microporous UiO-type MOFs were ozonated, and CAT was then used to encapsulate mesopores. In H_2_O_2_, MOF engines undergo an enzymatic reaction, which produces oxygen bubbles to propel the motor.

#### 2.3.3 Multienzymes

Because urease, CAT, and glucose oxidase (GOx) are the three most often used biological enzymes, multienzyme-driven nanomotors can be used to synthesize various biocompatible enzymes into a micro/nanomotor that functions as a biocatalyst to produce propulsion when substrate fuels are present.

Because the use of enzymes to propel micro/nanomotors requires fuel (water, hydrogen peroxide, urine, glucose, *etc*.), multienzyme-driven micro/nanomotors are necessarily fuel-dependent. Previously, a hybrid micro/nanomotor has been synthesized using CAT and urease 108. Biotin-streptavidin ligation was used to functionalize polystyrene microparticles with both urease and CAT. Particle diffusion increased with increasing matrix concentration, and the diffusion of CAT- and urease-coated particles also increased with increasing substrate concentration. Not only can urease and CAT be mixed, but CAT and GOx are also another common combination. Gao* et al*. designed a carbonaceous nanoflask (CNF) motor (**Figure 4C**) that moves spontaneously in glucose. The motor power was provided by the cascade reaction between GOx and CAT 109. By controlling the nanomotor's surface wettability, the micro/nanomotor can move in a certain direction. To enable propulsion, a hydrophobic CNF motor was developed to move from the round bottom to the opening neck (backward), and a hydrophilic version was developed to move from the opening neck to the round bottom (forward). In a different study, scientists developed a core-shell nanomotor exhibiting dual-enzyme functionality (designated as UTZCG) 110. The nanomotor featured a metalorganic NP framework that enhanced the complementary effects of starvation and photodynamic therapies. In the catalytic cell, glucose was decomposed by modified GOx to starve cells, and H_2_O_2_ was produced. Subsequently, CAT catalyzed H_2_O_2_ decomposition, leading to propulsion.

In nanozymology or nanomaterial catalysis 111, enzyme-catalyzed reactions play a facilitating role 112. In living organisms, enzymes convert chemical to mechanical energy and facilitate other biochemical reactions. The application of enzymatic biocatalysts to power micro/nanomotors is a novel approach. Enzymatic reaction fuels are more ecofriendly and degradable than traditional fuels, and enzyme-powered micro/nanomotors show potential for biomedical application breakthroughs owing to their biocompatibility. However, enzymes have several drawbacks; for instance, although CAT-driven EMNMs can move rapidly owing to bubble propulsion, hydrogen peroxide is toxic to living cells. Various EMNMs can be driven by urease, CAT, and several other enzymes because key EMNM materials can perform on-demand tasks. The more common biological enzymes are urease, CAT, and GOx, which are found widely in nature, not only in animals but also in plants. Although many enzymes exhibit the same inherent properties that could enable the enzymes to be used as motors for autonomous movement, most enzymes exhibit limitations that hinder the control of their motor behavior, which limits their practical application. Clearly, natural EMNMs are not designed to accomplish the intricate operations or specific tasks required for guided locomotion. To be useful, enzyme-driven micro/nanomotors must improve their propulsion and overcome various limitations in complex environments. Currently, the methods most used to improve the driving force are the improvement of the enzyme catalytic ability by modifying the motor shape to accommodate hydrodynamics (e.g., improving the enzyme catalytic unit) and the utilization of cascade reactions with two or more enzymes. A combination of more advanced and intelligent testing or analytical techniques will be required to elucidate the energy conversion mechanisms of enzymatic reactions and generate the driving force. This would provide a theoretical basis and strategic guidance for subsequently studying EMNMs exhibiting more advantageous propulsion capabilities 96.

### 2.4 Living cells

The design, propulsion, and application prospects of cellular micro/nanomotors have considerably advanced 113. To develop synthetic micro/nanomotor applications that can function in biological systems without causing harm, biocompatible and biomimetic micro/nanomotor devices are required 114. Researchers have developed different artificial micro/nanomotor cells that imitate micro/nanomotors. Synthetic micro/nanomotors can be functionalized with various cellular elements, such as membranes, to assume cellular traits 115-117. Flagella, such as those on sperm, synthetic bacteria and MTB, inventive magnetic filaments, and bacteria-driven microrobots, are used for low-Reynolds-number propulsion 118.

#### 2.4.1 Sperm

**Figure 5:**
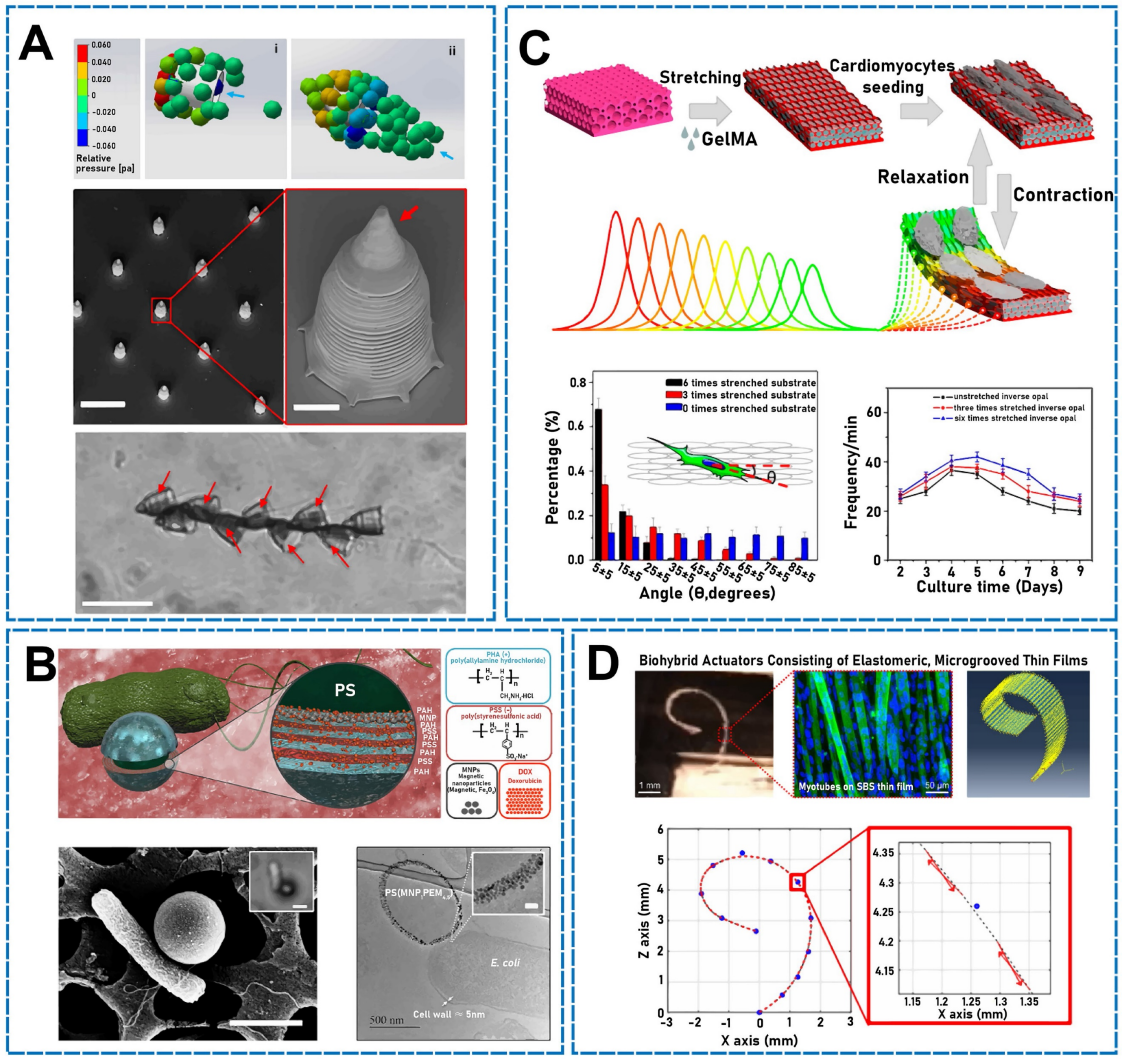
(A) Hydrodynamic simulations of the model. SEM images of streamlined-horned caps. Sperm train swimming in sperm medium. Reproduced with permission 123. Copyright 2020, American Chemical Society. (B) The design of multifunctional bacteria-driven microswimmers. SEM image of a single PS (MNP_1_PAH/PSS) _4_PAH-attached cell. TEM image of a microswimmers thin section. Reproduced with permission 125. Copyright 2017, American Chemical Society. (C) Synthesis of the biohybrid actuator. Cardiomyocytes drive corresponding color changes in substrates structures. Orientation angle frequency distribution of cardiomyocytes. The beating properties of cardiomyocytes. Reproduced with permission 133. Copyright 2019, American Chemical Society. (D) Biohybrid machine model. Finite element model simulation setting and results. Reproduced with permission 143. Copyright 2019, American Chemical Society.

In the 1950s, Machin emphasized that sperm motility was not accounted for or explained by forcing in the cell membrane but by motors distributed along the flagellum 119. Magdanz* et al*. combined microtubules and single motile cells to develop a microbiorobot that could be navigated to a prescribed target location 118. The authors used bovine sperm cells because these cells are similar in size and shape to human ones. The sperm cells entered microtubules and resided in tube cavities. Spermatozoa exhibited very powerful movements and interacted with magnetic microtubules to enable forward movement. Because the bovine sperm cells functioned as the driving force in the hybrid system, autonomous free-swimming functionalized sperm micromotors (FSFSMs) were developed 120. Natural sperm biomotors have been functionalized using various synthetic nanopayloads, such as endocytosis-particle-modified fluorescein isothiocyanate Pt NPs, CdSe/ZnS quantum dots, and doxorubicin-hydrochloride-coated iron oxide NPs. Under various biological and environmental conditions, FSFSMs exhibited highly effective self-propulsion, and chemoattractant-induced clustering was controlled. The FSFSM velocity was controlled by changing the osmotic pressure of the solution, which changed the flagellum lengths. Similarly, Xu *et al*. used the powerful sperm propulsion as an engine to develop a sperm-mixing micro/nanomotor for precisely delivering drugs 121. The micro/nanomotor comprised a 3D-printed four-armed magnetic tubular microstructure and a motile sperm cell functioning as a power source and medication carrier, respectively. Furthermore, a quadruped exhibiting four flexible arching arms and a tubular body was developed. Polymer structures were designed using two-photon and 3D nanolithography. Then, a 10 nm iron layer was asymmetrically coated on the tetrapod microstructure inclined at 15° to generate easy axis magnetization. In addition, to improve the composite biocompatibility, a 2 nm layer of titanium was deposited. Previous studies have shown that when the applied arm force was approximately 128 pN, the corresponding displacement was approximately 116 nm, which was sufficient to release sperm 122. When 450 pN was applied, the displacement rose to 407 nm, which may be produced by hyperactivated spermatozoa (exhibiting an asymmetric beating pattern and larger tail amplitude). Sperm cells function as propulsion sources, while magnetic microstructures are used to guide and release sperm. Additionally, sperm can generate a strong propulsion force and perform rheotaxis/thigmotaxis to swim, which is why sperm and sperm micro/nanomotors have attracted considerable research interest for bloodstream applications. At the biological microscale, sperm cells are outstanding swimmers. Xu *et al*. developed a hybrid sperm micro/nanomotor (**Figure 5A**), namely, a cap exhibiting a horn-like structure that can not only be assembled with other microcaps for transporting multiple sperm cells but can also be used as an anchoring structure 123. Streamlined horned caps can maintain the position of sperm micro/nanomotors at rapid blood-flow velocities when sperm are forced toward the channel surface by an external magnetic field. The cap was designed to reduce micro/nanomotor resistance and enable the cap to squeeze past blood cells and through uneven liquids. The motor could actively swim (using continuous pulsatile actions) in the blood flow, and the sperm tiles provided a powerful driving force.

#### 2.4.2 Bacteria

Many bacteria swim in aquatic environments to acquire resources, disperse progeny, and infect hosts. In many bacterial species, motion is driven by a rotating flagellum, which is in turn driven by a bidirectional flagellar motor 124. At the micrometer scale, bacterial cells can efficiently swim utilizing food sources in their environment. Additionally, bacteria can use synthetic substances for various functions 125. Researchers have discovered that the *E. coli* envelope can be disrupted and recycled to form nanovesicles. The bacterial envelope protects bacteria from external and dangerous environments 126. Bacteria are a good example of an elegant and fully self-sufficient natural machine, which swims efficiently, senses the surroundings, finds food, and communicates with other members of the same class. Therefore, to design and perfect functions for application to artificial microdevices, researchers have developed micro/nanomotors that imitate bacteria or directly apply their functions 7.

The attachment of bacteria to the surface of synthesized microparticles is a critical parameter for preparing bacteria-driven microswimmers. To exploit bacteria's excellent and robust autonomous motility and inherent chemotactic behavior as intelligent carriers, scientists have combined bacteria with polymeric beads 127. Park* et al*. developed a multifunctional, high-performance, bacteria-driven microswimmer (**Figure 5B**) 125. The main constituents were single *E. coli* cells, which adhered to the surface of drug-loaded, MNP-embedded, multilayered polyelectrolyte microparticles. The authors found that tuning the viscoelastic characteristics of bacterial surface interactions may be vital for microswimmer movement and bacterial attachment. In addition, the biohybrid microswimmers exhibited directional motion and skewness under magnetic guidance and chemoattractant gradients, respectively. Similarly, because *E. coli* was a key material for propelling hybrid micro/nanomotors, researchers used red blood cells (RBCs; erythrocytes) to construct bacteria-driven microswimmers that could be guided by bacteria *in vitro* 128. RBCs loaded with the anticancer drug doxorubicin and superparamagnetic iron oxide NPs (SPIONs) were attached to bioengineered motile *E. coli* MG1655 through a biotin-avidin-biotin binding complex to form a multifunctional biohybrid microswimmer. The bacteria provided autonomous on-board propulsion to biohybrid microswimmers, and SPION-loaded RBCs enabled external magnetic navigation. In another study, the nonpathogenic MTB *Magnetospirillum gryphiswaldense* was combined with drug-loaded mesoporous silica microtubules to construct controllable microswimmers that could deliver antibiotics to infectious biofilms 129. The movement of the hybrid organisms was also studied under the exogenous stimulation of a magnetic field. Vincenti* et al*. designed a rotary motor comprising living biological entities driven by an external alignment field 130. At higher bacterial concentrations, interactions resulting from bacterial swimming activity in these regions formed a collective solid-like vortex flow in the central droplet core. To fabricate a similar hybrid structure, MTB had to accumulate in certain regions in a uniform magnetic field. The congregation of this active swimmer was limited by crowding, which led to instability that triggered coordinated movements at the droplet scale. Furthermore, the dependence of the active magnetic fluid on the original properties was the key, and the surrounding fluid was torqued by any swimming kinematics that changed the direction of the bacterial magnetic moment relative to that in an applied magnetic field.

#### 2.4.3 Cardiomyocytes

The heart's pumping function is driven by the contractile force of myocardial engines, and muscle cells (cardiomyocytes) act through collective contractile activity 131. In a previous study, researchers designed a polyacrylonitrile-based hydrogel yarn comprising uniaxially aligned nanofibers 132 and then implanted chicken cardiomyocytes in nanofiber hydrogel yarns to develop living cell-based microactuators. The devices maintained spontaneous cardiomyocyte pumping for 7 days. Shang* et al*. investigated an inverse opal exhibiting anisotropic periodic elliptical macropores and a hydrogel filling, stretch-derived for constructing and assembling tissues of biohybrid actuators (**Figure 5C**) 133. The researchers found that cultured cardiomyocytes induced into a highly ordered arrangement on the substrate surface could beat spontaneously and that spontaneous beating was recoverable. Cardiomyocyte beating was accompanied by cell contraction and elongation, and the synchronous cycle of deformation actuations that the anisotropic inverse opal can be observed as commensurate shifts in their photonic band gaps and structural hues. Cardiomyocyte-driven inverse opals offer the possibility for developing biohybrid actuators exhibiting self-driving capabilities. Inspired by the mechanism of structural color regulation in a chameleon, researchers developed a conceptually distinct structural color material by engineering the assembly of cardiomyocytes organized on a synthetic inverse opal hydrogel membrane exhibiting self-regulation ability 134. During cardiomyocyte beating, cellular contraction and elongation caused the substrate membrane structure to exhibit a cycle of morphological and volumetric changes. Inspired by the crawling mechanism of caterpillars and the movement of snakes, Sun* et al*. developed a soft biological robot comprising asymmetric claws and carbon nanotube-induced myocardial tissue layers 135. During cardiomyocyte contraction, the claws aided the directional movement. The directional conduction of the carbon nanotube layer adjusted the cardiomyocyte arrangement and improved the cardiomyocyte pulsatile and contractile properties. Cardiomyocyte-powered soft robots were excellent for simulating the caterpillar crawling behavior. In another study, researchers developed a tissue-engineered deformable robot that could assume different mechanical structures exhibiting different locomotive functions 136. The robot was powered by a muscular caudal fin that mimics the swimming of a whale and functions as a cellular engine powered by synchronically contracting heart tissue structures. The system could be optically triggered to change from deployment to retraction, which effectively changed the bending stiffness of the tail to minimize, or even shut down, its propulsive output.

#### 2.4.4 Insect dorsal vessels

Insect dorsal vascular (DV) tissue is environmentally stable and low maintenance, which make it ideal for application to microdrives. Akiyama* et al*. designed an insect muscle-powered autonomous microrobot (iPAM) driven by crustacean cardioactive peptides, which are neuroactive chemicals 137. The robot comprised an artificial frame and DV tissue and moved quickly, which represents an elegant example of a biohybrid microdevice. For effectively utilizing the DV tissue contractile force to move the iPAM, the molding frame was prepared based on a simulation using finite element analysis software. Moth larval DV tissues were assembled on the frame. Compared with the movement velocities of previously reported models, that of the current model considerably improved. The system exploited the spontaneous contraction of the insect DV tissue (DVT). Under culture conditions, DVT cells are more environmentally stable than mammalian tissues and cells. In another study, researchers developed a spontaneously moving polypod microrobot (PMR) by implementing DVT excised from an inchworm 138. The researchers assembled the entire DVT on an inverted two-row array of micropillars to develop a prototype, which moved spontaneously at 3.5×10^-2^ μm/s, and the contractile force of the entire DVT was calculated at 20 μN. From the prototype, they produced a real PMR, which exhibited considerably enhanced velocity and spontaneously moved at 3.5 μm/s. Uesugi* et al*. developed a bioactuator that exhibited contractile properties and controllability 139. The bioactuator was fabricated using the DVT of an insect (final-stage moth larva) and different stimulation strategies. The high robustness of the insect tissue produced an excellent biological actuator.

#### 2.4.5 Skeletal muscle cells

Skeletal muscles can generate stronger contractile forces than cardiomyocytes. Typical skeletal muscle cells can generate a force as strong as 400 μN 140. In animals, skeletal muscle is the primary actuator, which is the interface between neurons and bones. Owing to their modular structure, these muscles generate enormous contractile forces in rapid succession upon receiving signals from motor neurons. Skeletal muscles can be a key material for propelling micro/nanomotors and developing biohybrid robots 141. In all animals, pumps are critical parts. During the first moments of life, the tubular embryonic heart functions as a valveless pump, which generates unidirectional blood flow. After studying this basic pump, researchers designed a biohybrid valveless pump robot powered by engineered skeletal muscle 142. This pump robot comprised a soft hydrogel tube attached at both ends to a stiffer polydimethylsiloxane scaffold, which generated an impedance mismatch. A ring of contractile muscle tissue wrapped the hydrogel tube off-center and squeezed the tube with or without local buckling. Cyclic muscular contractions, electrically stimulated or spontaneous, further squeezed the tube, causing elastic waves to propagate along the hose and reflect back at the soft/hard tube boundary. The asymmetric placement of the muscle ring generates a wave-to-wave time delay that establishes a net unidirectional flow regardless of whether the tube is buckled. Presently, pump-bots can achieve flow rates of up to 22.5 μL/min. In animals, skeletal muscle cells are the main actuating factors and, with appropriate optogenetic modification, can be adjusted using electromagnetic or electric fields. Hasebe* et al*. designed a synthetic powerplant (**Figure 5D**) comprising microgrooved membranes powered by aligned contractile skeletal muscle cells 143. To develop the system, a thermoplastic elastomer comprising poly(styrene-*block*-butadiene-*block*-styrene) (SBS) was prepared. Because SBS thin films are easy to handle and fabricate and exhibit low flexural rigidity, they have attracted considerable research attention for application to flexible substrates that can be used for designing advanced biohybrid actuators that can deform when cells shrink. SBS thin films are an interesting candidate scaffold for developing biohybrid drives. Aydin* et al*. developed a biohybrid swimmer powered by an on-board neuromuscular unit 144. The swimmer's body comprised skeletal muscle tissue, independent soft scaffolds, and motor neuron-containing optogenetic stem cell-derived neural clusters. However, this sensory-motor pattern depended on the ability of neural units to direct muscle movement. Periodic muscle contractions generated by nerve stimulations drove the time-irreversible flagellar dynamics, which provided the driving force required for moving the micro/nanomotor tether forward.

Biohybrid microswimmers can have a pivotal impact on both tissue engineering and bionics 145. Cell-like micro/nanomotors (which can be categorized as cell-based and cell membrane-coated motors) have recently shown great progress. Cell-like micro/nanomotors, for which drivers exhibit biological components (e.g., bacteria or sperm drivers); synthetic micro/nanomotors (e.g., fuel-free actuation or chemical self-propulsion); or cellular micro/nanomotors (e.g., entire cells or cell membranes) can prevent biological contamination and facilitate micro/nanomotor propulsion in complex biological fluids, such as blood. Compared with static motors, cell-like micro/nanomotors can exhibit faster biological detoxification by designing and combining the dynamic motion characteristics of synthetic motors with the multifunctionality of cellular components and facilitate disease-targeted drug delivery. The integration and assembly of cell materials and artificial micro/nanomotor structures improve and prolong the actuation of biological fluids, drug loadings and releases, and localization and targeting of imaging and therapeutic drugs. Although these cell-like micro/nanomotors exhibit unique advantages, some important challenges remain for potentially applying such micro/nanomotors *in vivo*. These include the further development of improved propulsion in rich and complex biological fluids; collective propulsion to control the motor velocity and direction; comprehensive biocompatible size, design, and function; enhanced tissue retention in therapeutic and imaging applications; and abilities to eliminate residues through self-decomposition and consume toxic fuels (e.g., H_2_O_2_). To achieve long-term propulsion in blood, which is an important challenge in the application of micro/nanomotors, the cellular components must remain intact. Other cellular motors exhibiting intrinsic chemotactic behavior, such as sperm and bacteria, can be used for sensing and responding to chemoattractants and targeting cargo delivery *in vivo*. Furthermore, to apply cellular motors, such as bacteria, to organisms foreign to the host, attention must be exercised to prevent immune responses and infection 7. Sperm cells are a suitable candidate for working in physiological environments because sperm do not contain disease-causing proteins or multiply to proliferate colonies as other cells or microbes do. Bacterial biohybrids comprise self-propelled bacteria carrying micro/nanomaterials and can transport their payload to a specified location under exogenous stimulation conditions, which potentially opens avenues in minimally invasive medicine 146. Sperm-mixing micro/nanomotors exhibit good biocompatibility and potential applications in gynecological treatments, healthcare, and the detection of cancer or other diseases of the female reproductive system. However, sperm-mixing micro/nanomotors exhibit a short working life because living sperm cells are particularly prone to inactivation. Therefore, more research is required to develop cells that can replace sperm. Bacteria-driven microswimmers are multifunctional and high-performance micro/nanomotors. Bacteria can combine with synthetic substances to perform various functions. The translation of bacterial-driven microswimmers into cargo delivery and clinical diagnostic devices requires the conception and preparation of versatile cargo carriers exhibiting excellent properties, such as biocompatibility, payload efficiency, stability, biodegradability, and deformability 128. Cardiomyocyte beating is accompanied by cellular contraction and elongation, and cardiomyocytes exhibit pumping behavior in native myocardium tissue. Cardiomyocytes, insect DVT, and skeletal muscle cells all exhibit pumping behavior. Therefore, materials derived from these cells are inherently biocompatible, and micro/nanomotors synthesized from these cells do not elicit immune responses. Finally, autonomously moving cell-driven biohybrid motors would enable a built-in chemotactic motion, which avoids reliance on complex actuation equipment or harmful fuels 7.

## 3. Applications

The complex environment in which micro/nanomotors are positioned, primarily referring to endogenous and exogenous stimuli, cannot be isolated from the driving and application of micro/nanomotors. Endogenous substances more frequently include water 34, urine 35, glucose 36, and hydrogen peroxide 37, 38. In response to endogenous stimuli, micro/nanomotors produce a propulsion gradient. Micro/nanomotors can be used to propel living cells and biological organisms because water and hydrogen peroxide are relatively prevalent endogenous components in living organisms. One of the endogenous stimuli can function as both a micro/nanomotor driver and potential therapeutic agent. For instance, while driving the micro/nanomotors through redox interactions with essential components, endogenous stimuli can correct or improve hypoxic deficiencies in tumor microenvironments, which is crucial for implementing photodynamic therapy in cancer treatment. Optical 24-27, magnetic 28-30, auditory 31, 32, and electric 33 fields are other common external stimuli. Propulsion can be accomplished using magnetic fields, which can accurately modulate micro/nanomotors through the involvement and management of exogenous stimuli. This is possible because most materials of power devices fabricated using micro/nanomotors are magnetic substances 74.

In biomedical disciplines, self-propulsion micro/nanomotors are essential 147. Nanomotors exhibit different morphologies 148 and motion mechanisms 149, 150 and are fabricated using materials 151, 152 exhibiting different biocompatibilities 153-155. Owing to important advances over the past few decades, micro/nanorobots can efficiently navigate to target destinations in physiological environments by converting various energies to kinetic energy and attaining propulsion. According to previous studies, micro/nanomotors offer a wide range of applications, including the direct loading, transport, and delivery of therapeutic payloads to disease locations, which minimizes detrimental effects on organisms and enhances therapeutic results 13. Numerous facets of life, including biosensing and disease treatment, are impacted by employing nanomotors.

### 3.1 Biosensing

The trapping agent on the micro/nanomotor surface captures and detects environmental substances with a power device. Janus particles are frequently used in self-propelled optical nanoprobes, biosensors, and micro/nanomotors 156, 157. Micro/nanomotors efficiently detect mainly nucleic acids (DNA and RNA) 158, 159, cancer cells 160, bacteria 161, hormones 162, and proteins (such as pepsinogen 163, lysozymes 164, immunoglobulins 165, and cholesterol 166).

Because of their enormous potential in actuation systems and smart sensing, micro/nanomotors and biosensors show potential as effective tools for administering medication and for early diagnosis in precision medicine. Micro/nanomotors and biosensors exhibiting good biocompatibility have been constructed by optically binding living RBCs to waveguides with fiberoptic probes. Additionally, blood pH has been measured by monitoring the RBC waveguide's light transmission, which could help diagnose pH-related blood diseases 17.

**Figure 6:**
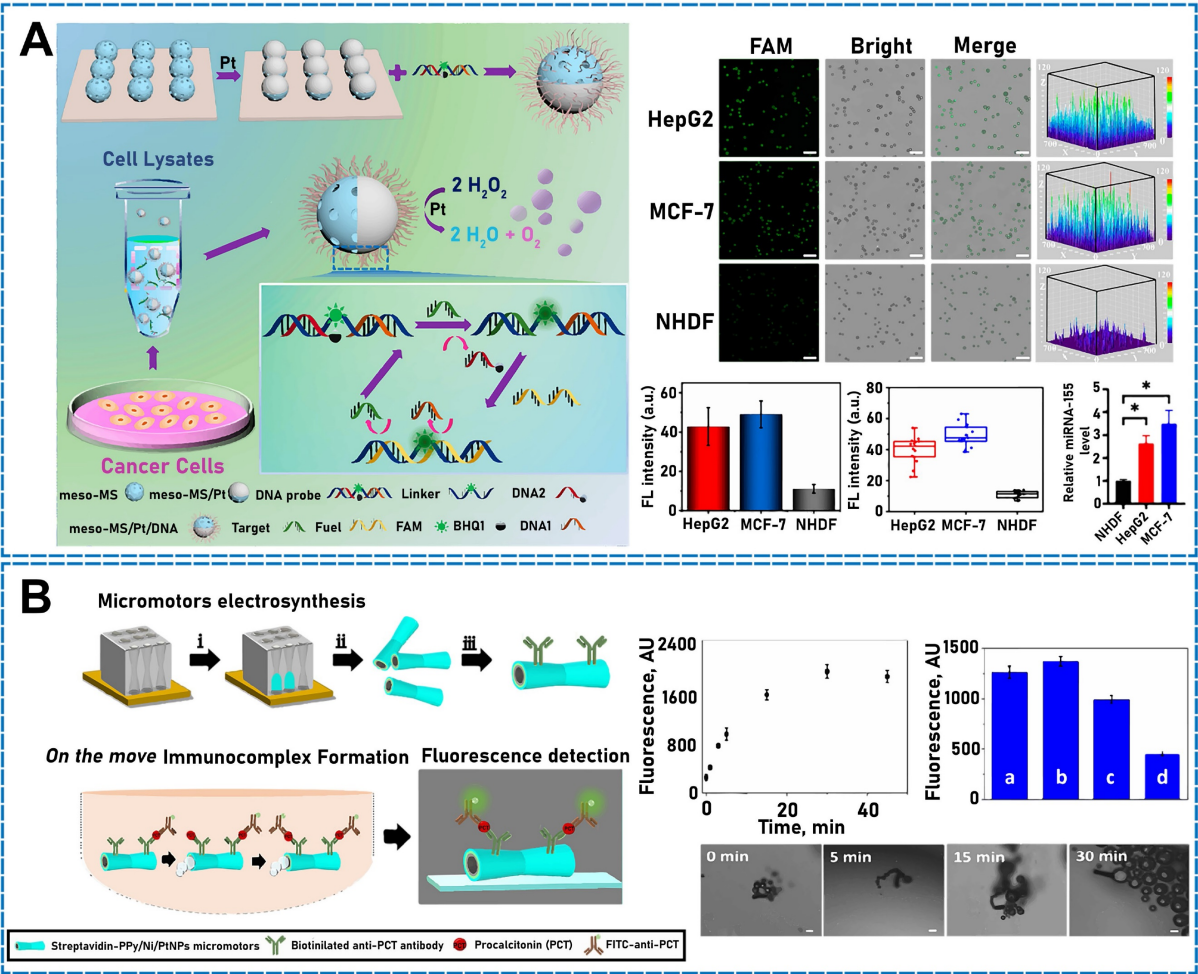
(A) Schematic diagram of Meso-MS/Pt/DNA micro/nanomotor for *in vitro* miRNA detection. Meso-MS/Pt/DNA CLSM images. FL intensity in NHDF, HepG2, and MCF-7 cell lysates. Relative miRNA-155 levels of NHDF, HepG2, and MCF-7 cells. Reproduced with permission 159. Copyright 2022, American Chemical Society. (B) Schematic diagram of the synthesis of anti-PCT-PPy/Ni/ PtNP micromotor. Determination of PCT by FMIm method. Effect of time on fluorescence signal and time-lapse images of the micromotor in plasma samples. Performance of immunoassay under different driving conditions. Reproduced with permission 161. Copyright 2020, American Chemical Society.

To mimic the mobility of planktonic jellyfish, Zhang *et al.* developed a micro/nanomotor comprising a multimetallic shell and catalase-modified DNA component 158. The jellyfish-resembling micro/nanomotor was propelled by the peroxidase's catalytic decomposition of H_2_O_2_ to generate oxygen. The open sensing surface of this micro/nanomotor effectively recognized the target molecule to detect DNA motion accurately and sensitively, which considerably benefits DNA detection. In addition, the micro/nanomotor is simple to prepare, exhibits good motion performance, and sensitively detects DNA motion. A class of endogenous small-molecule RNAs, known as miRNAs, plays a crucial role in cell differentiation, value addition, and death. The Janus mesoporous microsphere/Pt-based (meso-MS/Pt) nanostructure developed by Zhang *et al*. **(Figure 6A)** considerably improved target delivery and accelerated recognition for miRNA amplification and detection in intricate biological samples 159. Meso-MS/Pt/DNA micro/nanomotors have been demonstrated as a more creative concept and technique for analyzing miRNAs in authentic biological materials.

Procalcitonin (PCT) is an indicator used to determine the presence of bacterial infections. Calcitoninogen is a hormone generated by the human endocrine system to regulate calcium and phosphorus metabolism and lowers blood calcium levels for surveilling infectious diseases. To evaluate PCT and identify early sepsis, researchers have developed a fluorescence micromotor-based immunoassay (FMIm) (**Figure 6B**) 161. The micromotor actively recognizes PCT antigens based on magnetic guiding (Ni layer), the catalytic generation of oxygen bubbles (PtNP inner layer), and the high binding capacity of particular antibodies adapted to the polymeric polypyrrole outer layer. This FMIm enables the detection of an extremely low content (25 μL) in a clinically relevant concentration range (0.5-150 ng/mL) and direct PCT detection in clinical samples from very-low-birthweight infants suspected of having sepsis (LOD = 0.07 ng/mL).

Using a bispecific aptamer initiator, DNAzyme, and an entropy-driven circuit that uses rolling and walking motions to maximize signal amplification, Qi* et al.* developed a 3D DNA nanomotion biosensor for sensitively and specifically detecting lysozymes 164. Owing to the strong selectivity of the bispecific aptamer, the 3D DNA nanomotor biosensor may react to lysozymes with high specificity and quickly release the signal. Because the entire procedure is protease-independent, the operational stability is not affected by unfavorable environmental conditions. The lysozyme detection threshold was as low as 0.01 pg/mL in the linear range from 0.05 pg to 500 ng/mL. In addition, this strategy was highly accurate for analyzing samples, indicating the excellent application potential of the nanomotion biosensor for detecting nonnucleic acid targets.

To develop ultrasmall enzyme/photovoltaic nanomotors for detecting cholesterol, researchers prepared ultrasmall histidine-modified Fe_3_O_4_ NPs (UHFe_3_O_4_ NPs) that were connected directly with cholesterol oxidase (ChOx) 166. As a chemical catalyst, ChOx uses cholesterol oxidation to initiate UHFe_3_O_4_@ChOx and generate H_2_O_2_. UHFe_3_O_4_ NPs function as nanoenzymes to catalyze the subsequent color development reaction between H_2_O_2_ and 3,3′,5,5′-tetramethylbenzidine for detecting cholesterol while simultaneously functioning as a photothermal engine powering NIR light. These NPs exhibit both the peroxidase-mimetic property and photothermal effects. In the linear response range of 2-100 μM, UHFe_3_O_4_@ChOx functioned as a cholesterol sensor, which increased the sensitivity, accelerated the analysis, and detected cholesterol concentrations as low as 0.178 μM.

For accurate biological sampling, precision medicine to diagnose illnesses, and controlled delivery of medications, the various strategies of contemporary micro/nanomotor platforms offer a more practical method to ensure human life and health 167.

### 3.2 Disease treatment

In relation to human health, cancer remains a major challenge 168. The micro/nanomotor and power unit sizes enable the enhanced penetration of micro/nanomotors in sexual organs and therapeutic tissues, which provides micro/nanomotors with a wide range of application prospects in disease treatment. Nanomotors can be applied to treat malignant tumors 169, 170, 171, gynecological cancer 121, gastric ulcers 172, and atherosclerosis (AS) 173; capture and transport fertilized oocytes 174; eradicate fungal infections 175; treat bladder cancer 176; modulate the immune system 177; and repair damaged tissues 178, 179, and enhance bioadhesion and tissues penetration of biomedicine 180, all of which are extraordinarily important. Nanomotors can achieve these remarkable medical functionalities because they can provide targeted drug delivery 181,182 related to their driving mechanism and key materials.

To enhance synergistic antifungal therapy for transdermal delivery, researchers have designed a parachute-like nanomotor loaded with miconazole nitrate (PNM-MN) propelled under NIR irradiation 175. Owing to the distribution asymmetry in the PNM space, effective self-thermophoretic propulsion was achieved after the generation of a thermal gradient under NIR laser irradiation. The photothermal effect and pharmacological therapy of PNM can obliterate *Candida albicans* and biofilms, respectively. Furthermore, the drug uses nanomotors and NIR laser irradiation to penetrate the skin and reach the infection site effectively. Because the cellular membrane defends the cytoplasm, the delivery of extracellular drugs to cells is difficult, which limits their application to disease treatment. This encourages the application of self-propelled micro/nanomotors as carriers of orally administered drugs for treating gastrointestinal diseases. Researchers have developed a bioinspired, enzyme-driven biopolymeric micro/nanomotor that replicates the ability of *Helicobacter pylori* to penetrate stomach mucus 183. In acidic environments, immobilized urease effectively converts urea to ammonia, and the resultant increasing pH changes the local mucin layer from a gel to a sol, which aids the penetration of the gastric tissue layer. According to the study findings, the micro/nanomotors were eliminated from the body within 3 days without negatively affecting the digestive system. The enhanced penetration and retention of micro/nanomotors as an active oral delivery vehicle in the stomach will be used for treating various gastrointestinal disorders.

**Figure 7:**
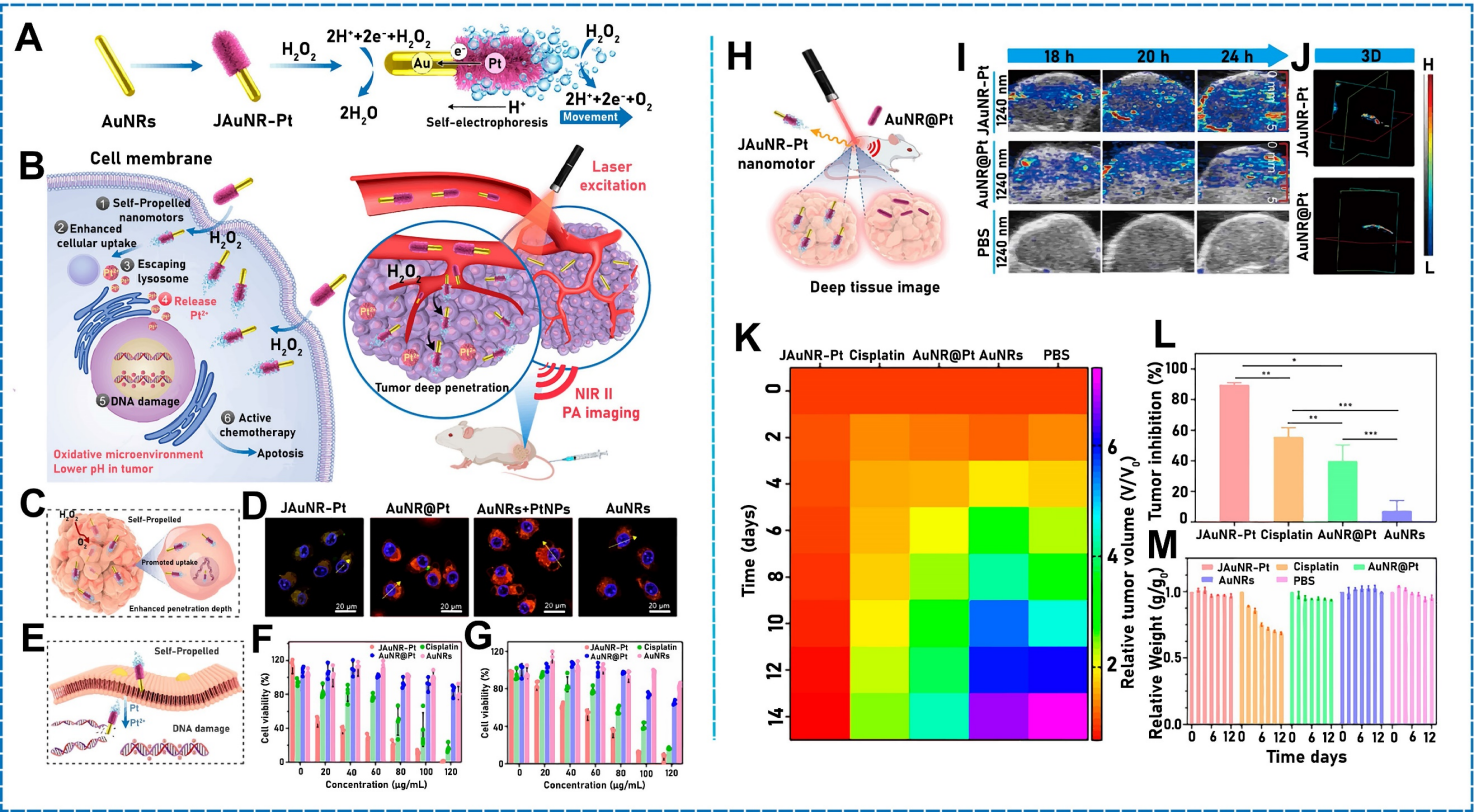
(A) Schematic diagram of the synthesis of JAuNR-Pt nanomotor. (B) JAuNR-Pt nanomotor for cancer therapy. (C) Schematic diagram of the permeability mechanism of the nanomotor. (D) Colocalization of nanomotors with the nucleus and lysosomal tracker. (E) Schematic diagram of cell treatment with JAuNR-Pt nanomotors. (F) - (G) Cell viabilities. (H) Schematic diagram of nanomotor permeability *in vivo*. (I) Photoacoustic (PA) image of *in vivo* tumor injected with JAuNR-Pt nanomotor. (J) 3D PA image of *in vivo* tumor. (K) Changes in tumor volume after *in vivo* antitumor. (L) Tumor inhibition. (M) Weight changes of mice in different treatment groups. Reproduced with permission 186. Copyright 2022, American Chemical Society.

In another study, an NO-driven nanomotor was constructed in a tumor microenvironment 184. During the movement, NO was released and played an important role in regulating the infiltration activity and behavior of T cells in the tumor microenvironment. The micro/nanomotor was accompanied by the release of NO during propulsion. The regulation of the tumor microenvironment by NO-driven nanomotors enabled the simultaneous deep penetration of microscale T cells and nanoscale drugs. Moreover, for simultaneous molecular imaging with spatiotemporal resolution, drug administration, and multifunctional cell targeting, Park* et al*. developed a mesoporous silica nanosphere exhibiting an optical nanocrescent antenna 185. This molecule was light driven, which enabled the rapid apoptosis of breast cancer cells through the delivery of doxorubicin. The excellent permeability of the power unit and micro/nanomotors make them promising candidates for delivering drugs and deeply treating malignant tumors. Li *et al*. developed an H_2_O_2_-propelled Janus gold nanorod-platinum (JAuNR-Pt) nanomotor (**Figure 7**) 186 for application to photoacoustic imaging in the second NIR region (NIR-II) of deep tumor tissues and effectively treated tumors with excellent detection and therapeutic integration. The JAuNR-Pt nanomotor driver enhanced the cellular uptake of the drug and improved the lysosomal escape, which was more conducive to the sustained release of cytotoxic Pt^2+^ ions in the nucleus to damage DNA and stimulate tumor cell apoptosis. The results suggested that the JAuNR-Pt nanomotor deeply penetrated, accumulated in, and treated tumors effectively.

Because sperm cells are naturally optimized to swim in the female reproductive system, they can be applied for treating cervical cancer and other gynecological diseases. Therefore, Xu* et al*. developed a drug delivery system based on sperm-hybrid micro/nanomotors 121. In this bionic micro/nanomotor, sperm naturally swim to tumor lesions and fuse with somatic cells therein, which enables them to function as drug carriers for potentially treating cancers in the female reproductive tract by effectively transferring drugs to target cells/tumors. In addition, sperm cells function as a propulsion source, while the magnetic microstructure is used to guide and release sperm. The microstructural arm bends as it contacts the cell, which generates a pathway for releasing sperm. This sperm-hybrid micro/nanomotor exhibits excellent biocompatibility and has potential for application to gynecological care and the treatment and detection of cancer and other diseases in the female reproductive system.

Currently, several drugs exhibit low efficacy for treating bacterial infections, which have become multidrug-resistant. This has broadened the scope of bacterial infections and, therefore, endangers human health. To solve this problem, researchers have designed nanomotors 187 comprising urease-, lysozyme-, and urease- and lysozyme-functionalized MSNPs (U-MSNPs, L-MSNPs, and M-MSNPs, respectively) exhibiting antibacterial properties against nonpathogenic planktonic *E. coli*. Urease catalyzed the urea generation of NaHCO_3_, NH_3_ drove the micro/nanomotors, and U-MSNPs exhibited the highest bactericidal activity. Above 200 μg/mL, U-MSNPs reduced the biofilm biomass of uropathogenic *E. coli* by 60%. These results demonstrated the ability of functionalized enzymatic micro/nanomotors to fight infectious diseases.

**Figure 8:**
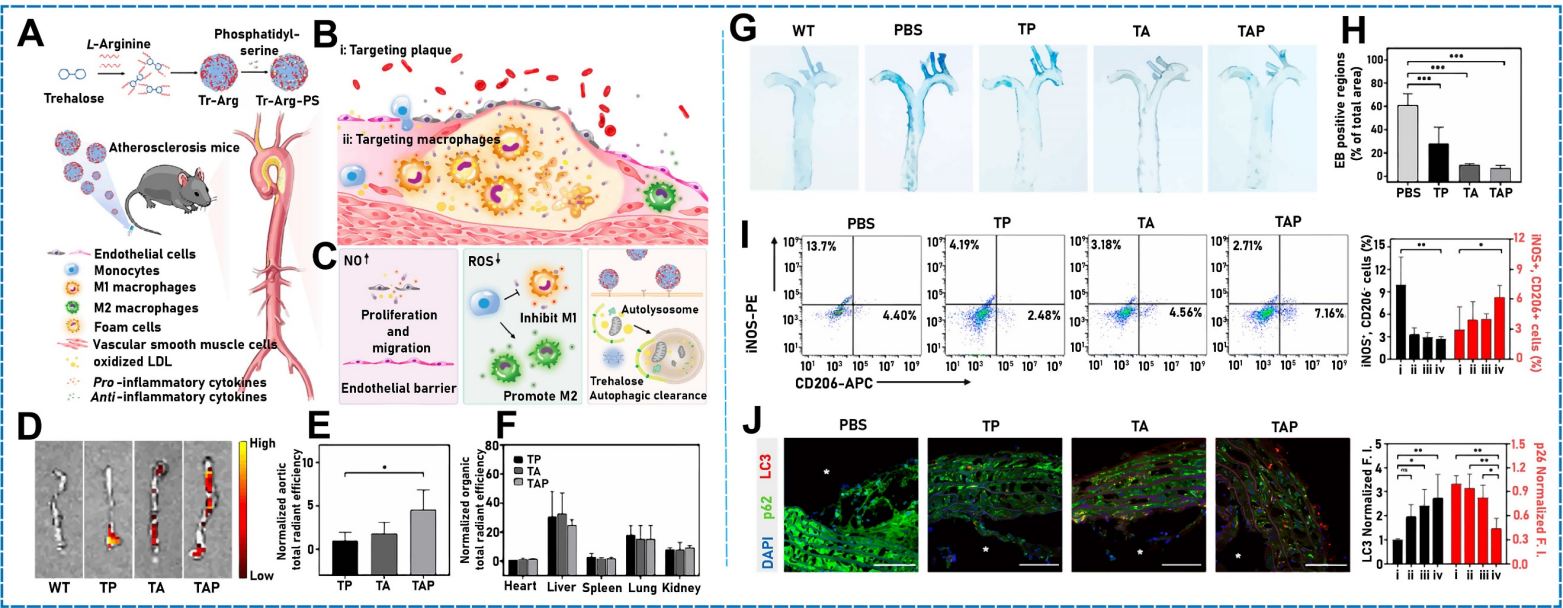
(A) Preparation process of TAP nanomotors. (B) Targeting mechanism of TAP nanomotors. (C) Integrated antiatherosclerosis effect of TAP nanomotors. (D)-(F) Capacity to target plaque-associated macrophages *in vivo* and anti-atherosclerotic response. (G) Aortic staining of atherosclerotic mice with different sample treatments. (H) Quantitative analysis. (I) Analysis of macrophage subsets by flow cytometry in the aortas of mice with atherosclerosis. (J) Autophagy-related proteins are seen in cryosections of the aortic arch in immunofluorescence pictures. Reproduced with permission 188. Copyright 2022, American Chemical Society.

Traditional methods for treating AS involve surgical and pharmaceutical interventions. However, these methods are invasive and cause various side effects. Owing to the micro/nanomotor's permeability and drug delivery capability, considerable progress has been made in alternative methods for treating AS. Wu* et al*. developed a driver for alginate (Tr, one of the mTOR-independent autophagy inducers), L-arginine (Arg), and phosphatidylserine (PS) **(Figure 8)** 188. At AS sites, ROS and inducible nitric oxide synthase (iNOS) were highly expressed and functioned as agents for inducing the chemotactic behavior of micro/nanomotors, which is the first step in targeting AS plaques. Then, corresponding PS signals were used to target macrophages in AS plaques gradually and precisely. During the treatment, ROS regulated the M2 polarization of macrophages, and NO contributed to the reconstruction of the endothelial barrier, which can treat AS in a multilinked manner.

We have highlighted the “smart” behaviors of micro/nanomotors for various fantastic biomedical applications 189. Owing to their ability to distribute medications, treat various ailments, and investigate intricate biological microenvironments without using any external cells, nanomotors are obviously indispensable.

## 4. Conclusions and perspectives

To date, micro/nanomotors have shown excellent promise in disease treatment and biosensing applications. Metals or compounds being not the only key materials available for driving micro/nanomotors, biocompatible, environmentally friendly, and biodegradable “biosmart” micro/nanomotors have been developed by combining various preparation techniques and continuously exploring key materials for driving micro/nanomotors. Enzymes, which are abundant in living organisms and transform chemical into mechanical energy, are another example of a key material. Biocompatible and biodegradable micro/nanomotors can be produced using enzymes as biocatalysts to provide the energy required to drive such motors. Owing to their natural propulsion or contractility, spermatozoa, cardiac skeletal muscle, and insect dorsal blood arteries can all be employed as “pumps” for biohybrid micro/nanomotors. In addition to exhibiting well-controlled deformability, micro/nanomotors can reduce biological contamination and facilitate micro/nanomotor movement through complex biological fluids, such as blood. The investigation of these drivers may suggest further avenues for applying micro/nanomotors.

Because several important and intriguing studies support the application expansion of micro/nanomotors, a more thorough overview is required because, to the best of our knowledge, no research is currently available in the literature on the classification of micro/nanomotors according to different material types, endogenous substances, and exogenous stimuli, especially for micro/nanomotors powered by cardiomyocytes, insect dorsal vessels, and skeletal muscle cells. Additionally, our in-depth analysis of micro/nanomotors may bring readers up to date on the development of these interdisciplinary fields, which include chemistry, biology, nanomedicine, and materials research. Because micro/nanomotor research is a multidisciplinary field, the limitations of micro/nanomotors will be overcome, which will improve the practical application prospects of micro/nanomotors.

In this review, we have summarized the main components powering micro/nanomotors and examined the effects of endogenous compounds and external stimuli on micro/nanomotor movement. We also explored the application of micro/nanomotors for biosensing and treating diseases. Because they are tiny in liquid environments, micro/nanomotors cannot overcome Brownian motion, which hinders the generation of more ideal directional motion, and different resistances in complex environments are some of the main drawbacks of micro/nanomotors. Additionally, whether micro/nanomotors can meet all the challenges for developing desirable practical applications remains unclear. However, compared with conventional nanomedicines, micro/nanomotors face numerous challenges related to shorter lifespans, more difficult preparation techniques, high costs, and potentially unfavorable biocompatibilities. Currently, micro/nanomotors may be superior to conventional nanomedicines for treating malignant diseases. Compared with conventional nanomedicines, micro/nanomotor power devices may effectively, rapidly, and completely detect and decompose targets in real time and be recycled and reused. All these characteristics endow micro/nanomotors with excellent application potential. To improve therapeutic outcomes, micro/nanomotors should boost synergistic treatments, deeply penetrate targeted tissues, effectively decompose targets, and mitigate the negative effects of extremely toxic medications.

The utilization of these essential materials to drive micro/nanomotors for sensing detection, illness treatment, and other intriguing applications must still be developed further and should be thoroughly evaluated in practical and clinical applications. The key components that power micro/nanomotors are currently being intensively studied and analyzed in a wide range of applications. The application potential of biosensing, which should provide insights for transmitting biosignals, is unquestionably beneficial for improving health. In the meantime, potential micro/nanomotor applications extend beyond sensing and detection and include the treatment of severe illnesses. Micro/nanomotors enable synergistic hydrogen chemotherapy and drug delivery for treating cancer and have considerable promise for numerous biomedical applications, including platforms and methods for treating various malignancies.

## Figures and Tables

**Table 1:** A summary of the properties of micro/nanomotors according to the key materials driving them.

Key material		Particle size	Motion speed	Movement mechanism	Application	Advantage	Ref.
Metallic nanocomplexes	Pt	~20.28 ± 7.55 µm (D)	265.52 ± 41.85 µm/s	Bubbles propulsion	Improved the Cs adsorption	-	48
	Mg	10-25 µm (D)	~127.3 μm/s	Bubbles propulsion	Fulfill diverse biological tasks	-	52
	Zn	8.9 ± 0.7 µm (D)	203.5 μm/s	Bubbles propulsion	Target treatment of bacterial infections	-	59
	Au	67.8-80.6 nm (D)	~1.46-6.44 μm/s	Self-thermophoretic force	Recognizing cancer cells	-	60
	Metallic compounds (MnO_2_)	~2 μm (ID) ~5 μm (D) ~10 μm (L)	77.2-466.4 μm/s	Bubbles propulsion	-	Low cost, wide fuel concentration range, easiness of fabrication, tunable crystal structure and surface morphology, controllable size and geometry, high efficiency and long lifespan.	10
	Multiple metals (Au/Pt)	62 ± 3 nm (D)	20.4 μm/s	Concentration gradient	Drug-molecule cargo loading	The motion-behavior endowed efficient cellular uptake of micro/nanomotors.	72
Nonmetallic nanocomplexes	Inorganic nonmetallic compounds	4.0 mm (D) 6.0 mm (L)	14.78 ± 4.82 mm/s	Bubbles propulsion	Targeted bactericidal effect	-	80
	Organic compounds (HLA_n_)	~120-385 nm (D)	3-13 μm/ s	Bubbles propulsion	Promoting revascularization and anti-tumor	Zero-waste and self-destroyed nanomotors.	85
Enzymes	Urease	-	~4.04 μm/s	Concentration gradient	-	-	101
	Catalase	13.5 μm (L) ~3.0 μm (ID)	420 ± 18 μm/s.	Bubbles propulsion	Detect a variety of biomolecules	Good selectivity and reproducibility, excellent stability and assay performance.	102
	Multi-enzymes	41 ± 2 nm (D)	-	Bubbles propulsion	-	Synergistically promote the efficacy of the enzyme- driven nanomotor, NIR light-triggered PDT and GOx-induced ST effects.	110
Living cells	Sperm	> 50 μm (L) ~ 5 μm (D)	76 ± 17 μm/s	The sperm flagellum	Magnetic guidance and cargo transport	Strong sperm propulsion, sperm rheotaxis, biocompatibility	123
	Bacteria (Escherichia coli bacterium)	-	22.5 μm/s	Bacteria-driven	Drug delivery	The bacteria can be integrated with synthetic substances to produce multiple functionalities through their biological actuation and sensing capabilities.	125
	Cardiomyocytes	~ 100 µm (THK)	-	Cardiomyocyte beating	-	-	135
	Insect dorsal vessels	150 μm (D)	3.5 μm/s	Insect dorsal vessel tissue contracting	-	Contracting autonomously and more environmentally.	138
	Skeletal muscle cells	0.5-11.7 μm (THK)	-	Skeletal myotubes	Biohybrid devices with predictable functionality	-	143

## Abbreviations

ALG: Alginate
AMB: Magnetospirillum magneticum AMB-1
AS: atherosclerosis
BiOI: bismuth oxyiodide
BNN6: N, N-di-sec-butyl-N, N-dinitroso-1,4-phenylenediamine
B@P@C: BNN6@PDA@CREKA
CAT: catalase
CCMP: calcium carbonate microparticle
CHI: chitosan
CNF: carbonaceous nanoflask
CREKA: Cys-Arg-Glu-Lys-Ala
Cu_2_O@GO: cuprous oxide @ graphene oxide
CXCL12: chemokine (C-X-C motif) ligand 12
D: Diameter
DV: dorsal vascular
DVT: dorsal vascular tissue
E-H-motor: emulsion hydrogel motor
EMNM: enzyme-powered micro/nanomotors
Fe-BTC: Iron 1,3,5-benzenetricarboxylate
FSFSMs: free swimming functionalized sperm micromotors
GO: graphene oxide
GOx: glucose oxidase
hQN: hairpin DNA quadrilateral nanostructure
ID: inner diameter
iNOS: inducible nitric oxide synthase
iPAM: insect muscle-powered autonomous microrobot
JMs: Janus micromotors
JMSNM: Janus mesoporous silica nanomotor
JRs: Janus rods
JUS motors: Janus upconversion nanoparticle-SiO_2_ micromotors
KF: kapok fiber
L: Length
LbL: layer-by-layer
L-MSNPs: MSNPs functionalized with lysozyme
LOD: limit of detection
meso-MS/Pt: mesoporous microsphere/Pt-based
miRNAs: microRNAs
M-MSNPs: MSNPs functionalized with urease and lysozyme
MNPs: magnetic nanoparticles
MOFs: metal organic frameworks
MOFtors: MOF motors
MSNP: mesoporous silica nanoparticle
MTB: magnetotactic bacteria
MΦ: Macrophage
NIR: near-infrared
NOS: NO synthase
NPs: Nanoparticles
PAzoMA: poly [6-(4-methoxy-4 -oxyazobenzene) hexyl methacrylate
PCT: Procalcitonin
PDA: polydopamine
PEDOT: poly(3,4-ethylenedioxythiophene)
PEDOT-PSS/Au: poly (3,4-ethylenedioxythiophene and sodium 4- styrenesulfonate)/Au microtube
PLGA: polylactic-glycolic acid
PMR: polypod microrobot
PPyNP: poly pyrrole nanoparticle
PS: phosphatidylserine
PSS: poly (sodium 4-styrenesulfonate)
PVA: poly (vinyl alcohol)
RBCs: red blood cells
RGO: reduced graphene oxide
ROS: reactive oxygen species
SAMTB: semi-artificial magnetotactic bacteria
SPION: superparamagnetic iron oxide nanoparticle
THK: thickness
3D: three-dimensional
UHFe_3_O_4_ NPs: ultrasmall histidine-modified Fe_3_O_4_ nanoparticles
U-MSNPs: MSNPs functionalized with urease
